# Comprehensive Mapping of the *Escherichia coli* Flagellar Regulatory Network

**DOI:** 10.1371/journal.pgen.1004649

**Published:** 2014-10-02

**Authors:** Devon M. Fitzgerald, Richard P. Bonocora, Joseph T. Wade

**Affiliations:** 1 Department of Biomedical Sciences, University at Albany, Albany, New York, United States of America; 2 Wadsworth Center, New York State Department of Health, Albany, New York, United States of America; Max Planck Institute for Terrestrial Microbiology, Germany

## Abstract

Flagellar synthesis is a highly regulated process in all motile bacteria. In *Escherichia coli* and related species, the transcription factor FlhDC is the master regulator of a multi-tiered transcription network. FlhDC activates transcription of a number of genes, including some flagellar genes and the gene encoding the alternative Sigma factor FliA. Genes whose expression is required late in flagellar assembly are primarily transcribed by FliA, imparting temporal regulation of transcription and coupling expression to flagellar assembly. In this study, we use ChIP-seq and RNA-seq to comprehensively map the *E. coli* FlhDC and FliA regulons. We define a surprisingly restricted FlhDC regulon, including two novel regulated targets and two binding sites not associated with detectable regulation of surrounding genes. In contrast, we greatly expand the known FliA regulon. Surprisingly, 30 of the 52 FliA binding sites are located inside genes. Two of these intragenic promoters are associated with detectable noncoding RNAs, while the others either produce highly unstable RNAs or are inactive under these conditions. Together, our data redefine the *E. coli* flagellar regulatory network, and provide new insight into the temporal orchestration of gene expression that coordinates the flagellar assembly process.

## Author Summary

Flagella are surface-associated appendages that propel bacteria and are involved in diverse functions such as chemotaxis, surface attachment, and host cell invasion. Flagella are incredibly complex macromolecular machines that are energetically costly to produce, assemble, and power. Flagellar production is tightly regulated and flagellar components are only synthesized when flagellar motility is advantageous. Regulation also ensures that flagellar components are produced in roughly the same order in which they are needed, increasing efficiency of the assembly process. The transcriptional regulation of flagellar genes has been studied extensively in the model organism *Escherichia coli*; however, many questions remain. We have used an unbiased, genome-wide approach to comprehensively identify all of the binding sites and regulatory targets for the two key regulators of flagellar synthesis, FlhDC and FliA. Our results redefine the flagellar regulatory network, and suggest that FliA binds many sites that are not associated with productive transcription. This work is important because it suggests possible new functions for FliA outside of the transcription of canonical mRNAs, and it provides new insight into the temporal orchestration of gene expression that coordinates the flagellar assembly process.

## Introduction

Bacterial flagellar synthesis and motility are highly regulated processes involving positive and negative input at the transcriptional, post-transcriptional, translational, and post-translational levels. This complex regulatory system allows for sequential production of flagellar components in roughly the order they are required for assembly. One of the primary ways this temporality is established is through the underlying hierarchical transcription network (reviewed [1]–[3]). Promoters for flagellar genes are divided into three categories (Classes 1, 2, and 3) based on their timing and mode of expression.

The atypical transcription factor FlhDC (more specifically, FlhD_4_C_2_) serves as the master flagellar regulator in *Escherichia coli*, *Salmonella enterica*, and other related enteric bacteria [4]. The *flhDC* operon is considered the sole Class 1 transcription unit. FlhDC expression and activity is regulated at a number of levels, allowing it to serve as an integrator of environmental, nutritional, and growth-phase signals [5]–[11]. In turn, FlhDC is responsible for activating the transcription, either directly or indirectly, of all structural and regulatory components of the flagellar machinery.

Class 2 promoters are directly activated by FlhDC and transcribed by RNA polymerase (RNAP) containing the primary σ-factor, σ^70^ [2]. Contacts between FlhDC and the carboxy-terminal domain of the α-subunit of RNAP are important for activation, but the precise mechanism is not known [12]. The seven commonly accepted FlhDC-dependent operons (*flgAMN*, *flgBCDEFGHIJ*, *flhBAE*, *fliAZY*, *fliE*, *fliFGHIJK*, and *fliLMNOPQR*) encode important regulatory factors and the structural components of the membrane-spanning basal body and associated export apparatus [1]. Notably, *fliA* encodes an alternative σ-factor [13]–[15] and *flgM* encodes its cognate anti-σ-factor [16]. The interplay between these two factors regulates the transition from early flagellar gene expression to late-stage gene expression.

When both FliA and FlgM are present in the cytoplasm, FlgM binds FliA, preventing interaction with RNAP, and repressing FliA-dependent transcription. Upon assembly of the basal body and secretion apparatus (from Class 2 gene products), FlgM is exported out of the cell, freeing FliA and allowing initiation of FliA-dependent transcription from Class 3 promoters. This coupling of transcription and assembly allows for efficient “just-in-time” expression kinetics, as has been described for some metabolic pathways [17]. FliA drives transcription of six commonly accepted Class 3 operons (*flgKL*, *fliDST*, *flgMN*, *fliC*, *tar-tap-cheRBYZ* (*meche*), and *motAB-cheAW* (*mocha*)) and can initiate transcription of its own operon, *fliAZY*. These Class 3 operons encode products needed in late flagellar assembly such as the subunits of the flagellar filament (flagellin), components of the motor, and chemotaxis-related regulatory factors. In addition to these classical Class 3 operons, FliA has been predicted or shown to drive transcription of genes involved in chemotaxis (*trg* [18] and *tsr* [19]), aerotaxis (*aer* [18], [20]), and cyclic-di-GMP regulation of motility (*yhjH* and *ycgR* [21]). FliA can also drive transcription of the Class 2 *fliLMNOPQR* operon [22], and FliA-dependent transcription of other Class 2 operons has been suggested [1], [2], [23].

In addition to their roles in the flagellar transcription network, FlhDC and FliA have been implicated in the regulation of non-flagellar genes. Studies have reported a host of non-flagellar genes and processes, such as cell division and anaerobic metabolism, regulated by FlhDC, or by FlhD alone [24]–[28]. However, the evidence provided for most of these additional target genes, such as changes in gene expression with no information on DNA binding, is far from conclusive. The reports of FlhD acting independently to regulate cell division have been directly refuted by another study [29] and many putative FlhDC targets fail to be repeatedly detected across multiple studies [20], [24], [25], [27], [28]. Furthermore, most previous studies fail to accurately distinguish between direct and indirect FlhDC-dependent regulation, as exemplified by the identification of the flagellar-related gene *aer* as an FlhDC target [25] when it is actually transcribed by FliA [18], [20]. There are a few well-characterized FliA-dependent promoters, such as *modA* [30] and *flxA* [31], that have no obvious function in flagellar synthesis or motility. Furthermore, there have been various bioinformatic studies that predicted FliA-dependent promoters based on sequence identity, finding hundreds of promoters, many with non-flagellar functions [30], [32], [33]; however, these predictions have not been tested experimentally.

Though the *E. coli* flagellar network has been studied extensively over the past few decades, many issues remain to be addressed. These include (i) the ability of FlhD to have regulatory effects independent of FlhC, (ii) the extent of co-regulation by FlhDC and FliA and the relative contributions of each at known complex promoters such as *fliAZY* and *fliLMNOPQR*, and (iii) the extent and composition of the non-flagellar regulons of FlhDC and FliA. Chromatin immunoprecipitation followed by deep sequencing (ChIP-seq) is a powerful technique to study the genome-wide localization of DNA-binding proteins. ChIP-seq provides high-resolution information about binding location and relative binding affinity *in vivo* [34]. Factors such as local DNA structure and the binding of nucleoid-associated proteins and/or other transcription factors affect binding and regulatory activity, making direct *in vivo* measurements incredibly valuable [35]. RNA-seq is a high-resolution method for assessing the transcriptomic differences between strains or conditions and allows for identification of novel transcripts such as non-coding RNAs [36]. By combining ChIP-seq and RNA-seq, one can assess the regulatory effect of each binding site and comprehensively establish which regulatory effects are direct and which are indirect. The combination of ChIP-seq and transcriptome analysis has recently been applied to many bacterial transcription factors [37]–[39] and σ-factors [40], [41]. This powerful combination of techniques has primarily been used to investigate single DNA-binding proteins, but can also be used to build global transcription networks [38]. In this study, we performed ChIP-seq on FlhD, FlhC, and FliA, and RNA-seq on motile wild-type, Δ*flhD*, Δ*flhC*, and Δ*fliA* derivatives of *E. coli* MG1655. These data reveal new binding sites and regulatory targets for both FlhDC and FliA, including the discovery of two FliA-dependent non-coding RNAs. We have comprehensively determined the direct and indirect regulons of these three proteins and demonstrate that this information can be used to build a detailed map of this hierarchical transcription network.

## Results

### Construction and validation of epitope-tagged strains

In order to perform ChIP experiments, we constructed strains in which FlhD, FlhC, and FliA were chromosomally epitope-tagged with a 3×FLAG tag. Since both termini of FlhD and FlhC have been implicated in complex formation or DNA-binding [42], [43], we inserted epitope tags into internal, unstructured regions (Figure S1A). FliA was tagged at the N-terminus. Tagged strains were constructed from a poorly motile MG1655 derivative, which contained no IS element upstream of *flhDC*. Spontaneous highly motile isolates were recovered following incubation on motility agar, as has been described previously [44]–[46]. To confirm that all isolates had gained motility through IS element insertion in the region upstream of *flhDC*, we sequenced the upstream and coding regions of each isolate (File S1). Each motile isolate had an IS element inserted in the region upstream of *flhDC* although the identity and location of the IS elements varied between strains (Figure S1B). No additional mutations were observed in *flhD*, *flhC*, or the region between *flhD* and *uspC*, in any of the tagged strains. To ensure that each of the IS element insertions was responsible for the motile phenotype of the strains, we constructed strains in which a selectable marker gene, *thyA*, was inserted in each observed insertion location. Insertion of the *thyA* cassette in each of the observed locations resulted in fully motile strains (Figure S1C). This experiment confirmed that while the tagged strains used in this study are not strictly isogenic, they are likely functionally equivalent. Additionally, it lends insight into IS element-associated motility by showing that motility acquisition is largely sequence- and location- independent. This is consistent with the previous hypothesis that IS element insertion up-regulates *flhDC* by displacing repressors bound in the upstream region [44], [45]. It is formally possible that the tagged strains contain additional mutations that confer motility, but this is extremely unlikely given the frequency with which motile strains were isolated.

**Figure 1:**
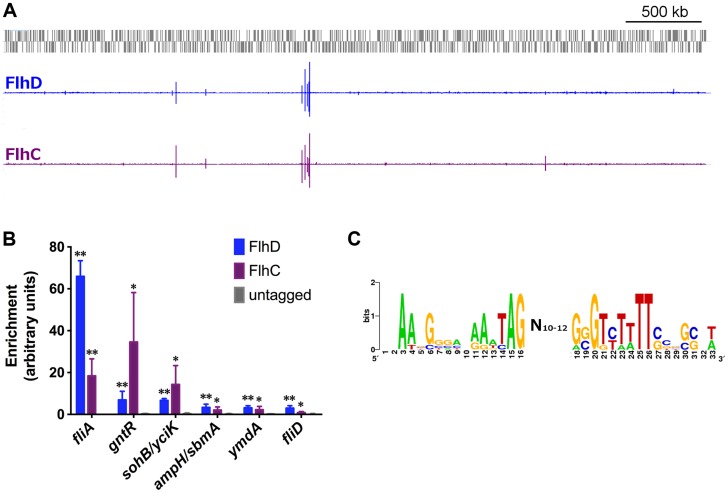
Genome-wide binding of FlhD and FlhC. (A) Genome-wide binding of FlhD (blue) and FlhC (purple) determined by ChIP-seq. Gray boxes represent genes in the MG1655 genome. (B) ChIP-qPCR enrichment at one well-known (*fliA*) and 5 novel FlhDC binding sites (n = 3). ChIP enrichment in tagged strains was compared to untagged control using two-sample T tests: * *p*<0.05, ** *p*<0.01 (one-tailed). (C) FlhDC motif derived in this study, using BioProspector [49] (score = 1.49) and visualized by inputing aligned sequenced into WebLogo [76].

While motile isolates were obtained for each tagged strain, all three tagged strains were less motile than a similarly selected wild-type strain, suggesting that epitope-tagging resulted in moderate functional impairment (Figure S1D). It is possible that the diminished functionality of the tagged proteins could prevent the identification of very weak binding sites; however, as discussed in detail below, the tagged proteins resulted in robust ChIP-seq signal at all extensively characterized binding sites and at many novel sites. Furthermore, the ChIP-seq signals generated for FlhD-FLAG and FlhC-FLAG proteins were nearly identical (Figure 1A) although the tags are inserted in distinct locations within the quaternary structure of the FlhD_4_C_2_ complex. This suggests that whatever the functional defect is, it is not influencing the ability of the tagged proteins to bind DNA. Finally, the RNA-seq experiments (performed with an isogenic set of strains) do not support the existence of any directly regulated targets not identified by ChIP-seq.

### Genome-wide binding of FlhD and FlhC

We used ChIP-seq to assess genome-wide binding of FlhD and FlhC in *E. coli* MG1655 grown to mid-exponential phase (OD_600_ 0.5–0.7) in Lysogeny Broth (LB). The genome-wide binding profiles of the two proteins are shown in Figure 1A. Based on a stringent peak-calling analysis, 10 FlhD binding sites and 8 FlhC binding sites were identified (Table 1). All 8 FlhC binding sites overlapped with FlhD binding sites. The 2 additional FlhD binding sites were also associated with substantial FlhC occupancy, barely missing the threshold in the peak calling analysis. None of the peaks identified in the FlhD or FlhC ChIP-seq experiments overlapped with regions enriched in control ChIP-seq experiments using untagged control strains. The complete colocalization of FlhD and FlhC binding is consistent with the proteins only binding DNA as a heteromeric complex. The 10 sites of FlhDC binding include all 5 binding sites associated with the 7 canonical flagellar Class 2 operons. An FlhDC binding site was identified upstream of *yecR*, a gene of unknown function, consistent with previous reports [20], [24]. The additional 4 binding sites identified represent novel FlhDC targets. Three of the novel FlhDC binding sites are in intergenic regions, upstream of one or more genes (*ampH*/*sbmA*, *yciK*/*sohB*, and *gntR*). The remaining novel binding site is located inside *csgC* and immediately upstream of a transcription start site for the adjacent gene, *ymdA* [47].

**Table 1:** FlhDC binding sites and expression of associated genes.

			Normalized gene expression^1^		
Peak Center^2^	FAT^3^ score (FlhD/FlhC)	Gene/Operon^4^	motile MG1655	Δ*flhD* 5	Δ*flhC* 5
395571	3/-	*ampH*	108	76	87
		*sbmA*	37	20	25
1104400	3/-	*(csgC)ymdABC*	(261)219	(1*)2*	(1*)3*
1130152	13/16	*flgAMN*	319	3*	3*
		*flgBCDEFGHIJ*	2664	9*	11*
1327204	4/5	*sohB*	77	154	147
		*yciK*	52	62	51
1964280	12/17	*flhBAE*	137	0*	0*
1986188	28/18	*yecR*	653	2*	1*
1999877	15/7	*fliAZY*	1257	1*	1*
2011110	20/10	*fliE*	418	2*	1*
		*fliFGHIJK*	719	3*	2*
2017573	38/27	*fliLMNOPQR*	509	1*	2*
N.D.	-/-	*fliDST*	633	3*	3*
3576809	2/7	*gntR*	160	140	127

1 Normalized gene expression values generated by Rockhopper. Expression values are for first gene in operon. Values in parentheses correspond with gene name in parentheses.

2 Peak centers represent an average of peak centers determined for FlhD and FlhC. Numbers represent genome coordinates relative to NC_000913.2.

3 Fold Above Threshold (FAT).

4 Gene(s) adjacent to binding site. Parentheses indicate an intragenic binding site.

5 Asterisks indicate significant differential expression (as defined in Methods) between motile MG1655 and indicated deletion strain.

To confirm FlhDC binding sites identified by ChIP-seq, targeted ChIP-qPCR was performed for all loci (Figure 1B; Table S1). Although ChIP-qPCR is not a completely independent validation method, it allows for analysis of more biological replicates, more straightforward comparison with untagged control strains, and reduces common artifacts enhanced by ChIP-seq library amplification [48]. Consistent with the ChIP-seq findings, FlhD and FlhC occupancy was significantly above that detected in an untagged strain (t-test, *p*-value≤0.05) for all loci. ChIP-qPCR was also used to evaluate FlhDC occupancy at previously predicted binding sites [24] not identified by ChIP-seq (Figure S2). The *fliDST* promoter was the only site to show significant occupancy by either protein (Figure 1B, Figure S2). This site was not identified by our ChIP-seq peak-calling analysis, but small, correctly shaped peaks are visible in the raw data for both proteins at this locus (see below). Hence, we consider the *fliDST* promoter to be a genuine FlhDC binding site and have included it in all downstream analysis, bringing the total to 11 FlhDC-bound sites.

The sequences surrounding the FlhDC binding sites identified with ChIP-seq and/or ChIP-qPCR were extracted and analyzed by BioProspector [49]. The resulting motif, found in 7 out of 11 sites, is low-scoring and degenerate (motif score = 1.49, Figure 1C) but corresponds well with previously reported motifs for FlhDC binding [20], [50].

### Direct and indirect regulation by FlhDC

**Figure 2:**
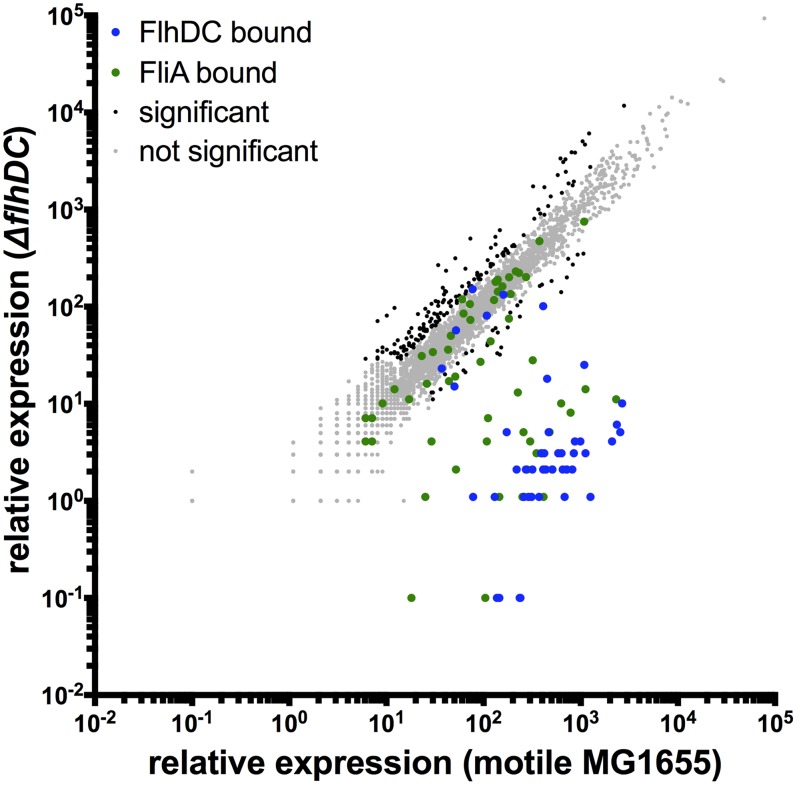
Genome-wide FlhDC-dependent gene expression. Relative expression of all genes in motile MG1655 versus relative expression in Δ*flhDC*. Gene expression values represent normalized expression values calculated by Rockhopper. Values on the y-axis represent the average of normalized expression values in the Δ*flhD* and Δ*flhC* strains. Color-coding indicates which genes are associated with FlhDC (blue) or FliA (green) binding. Genes not associated with FlhDC of FliA binding are color-coded according to whether they are significantly regulated (Rockhopper, q-value≤0.01) and changed at least 2-fold (black) or not (grey).

To determine the effect of the identified FlhDC binding sites on the transcriptome, RNA-seq was performed in the motile wild-type MG1655 strain and isogenic single-gene deletions of *flhD* and *flhC*. Differential gene expression analysis was performed using Rockhopper, an RNA-seq analysis program optimized for bacterial datasets [36]. Gene expression was almost identical in the Δ*flhD* and Δ*flhC* strains (Figure S3), as would be expected since the factors likely regulate the same genes and because the *flhD* deletion has a minor polar effect on *flhC* expression. Figure 2 shows a genome-wide comparison of normalized expression values in motile wild-type MG1655 versus the average normalized expression of the Δ*flhD* and Δ*flhC* strains. Overall, 228 genes were differentially expressed due to deletion of *flhD/flhC* (Figure 2). Of those significantly regulated genes, 40 are associated with FlhDC binding sites, indicating direct regulation.

**Figure 3:**
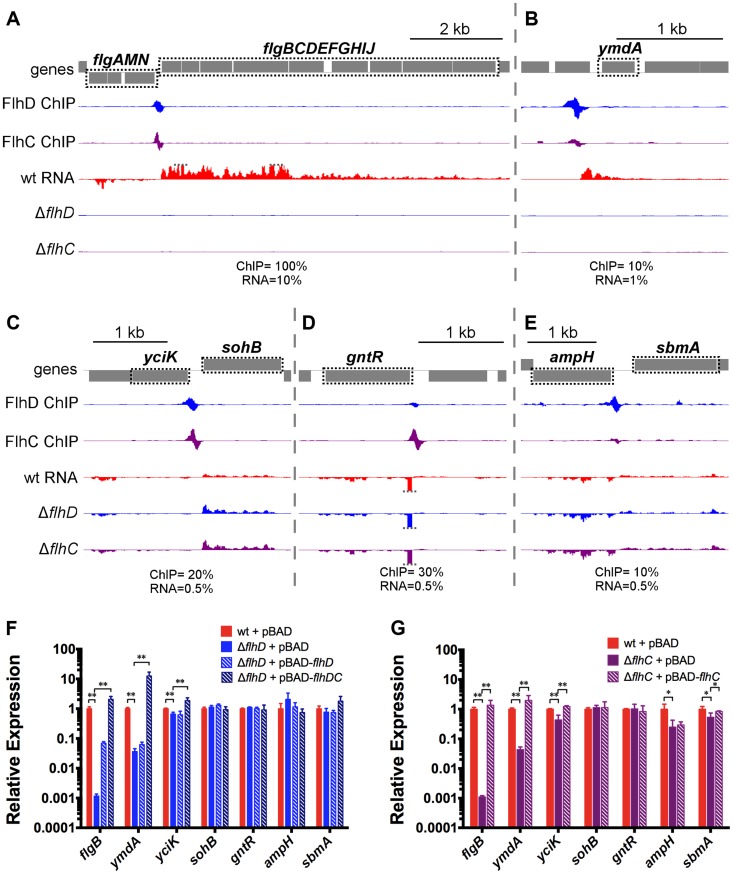
FhDC binding and regulation at known and novel targets. (A–E) Mapped reads from ChIP-seq and RNA-seq experiments. Genes and operons of interest are boxed (dotted black line) and labelled. Within each panel, both lanes of ChIP-seq data are scaled equivalently, and all three lanes of RNA-seq are scaled equivalently. Relative scales are indicated below each panel. Dotted gray lines indicate that mapped reads exceed the scale shown. (F & G) Gene expression, relative to *mreB*, measured by RT-PCR. Gene names are indicated on the x-axis and strain are indicated in the legend (n = 4–7). Statistical comparisons of were performed using two-sample T tests between the indicated groups: ** *p*<0.01 (two-tailed).

Of the 11 FlhDC binding sites identified by ChIP, 9 are associated with regulation of one or more adjacent genes (Figure 3A–E, Table 1). We detected strong, positive regulation of all previously reported Class 2 operons by FlhD and FlhC. Additionally, the RNA-seq data demonstrated FlhDC-dependent activation of *yecR*, *fliDST*, and the novel target operon *ymdABC* (Table 1, Figure 3B). The FlhDC binding site upstream of *sohB* seemed to repress expression by 1.95-fold (Figure 3C, Table 1). The RNA-seq experiments did not provide any evidence for regulation associated with the novel binding sites upstream of *ampH/sbmA* and *gntR* (Figure 3D–E, Table 1).

Selected examples of direct FlhDC-dependent regulation, or lack of regulation, were validated using qRT-PCR (Figure 3F–G). The presence or absence of regulation was confirmed for all genes tested, except *sohB* and the divergently transcribed gene *yciK*. While the RNA-seq data suggested that *sohB* was repressed 1.95-fold by FlhDC and *yciK* was not regulated, the qRT-PCR data clearly indicate the opposite: no regulation of *sohB* and significant, but slight positive regulation of *yciK* (1.5-fold for FlhD and 2.29-fold for FlhC; Figure 3F–G). Positive FlhDC-dependent regulation of *yciK* is also supported by complementation experiments. Small (less than 4-fold) but significant changes in *ampH* and *sbmA* levels are detected between wild type and Δ*flhC*; however, these changes are not detected in the Δ*flhD* strain or supported by complementation experiments (Figure 3F–G). RNA levels were also determined using qRT-PCR in a variety of complemented strains. As suggested by the motility assays (Figure S1E), *flhD* overexpression could only partially rescue the *flhD* deletion, but overexpression of *flhDC* restored target gene expression to wild-type or higher levels (Figure 3F). Overexpression of *flhC* alone in the *flhD* deletion strain did not rescue expression levels at all, demonstrating that the Δ*flhD* phenotype was not solely due to the polar effect on *flhC* expression (). The *flhC* deletion could be completely complemented by *flhC* overexpression, but target gene expression never exceeded wild-type levels, suggesting that the wild-type levels of *flhD* expression limit the possible pool of active FlhDC complexes (Figure 3G).

### Mechanism of direct regulation of transcription by FlhDC

**Figure 4:**
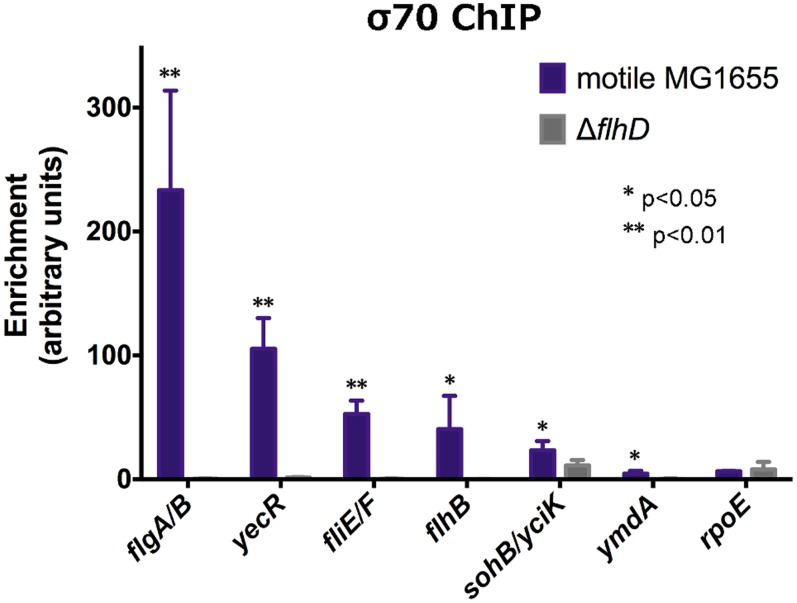
FlhDC is required for σ^70^:RNAP recruitment to FlhDC-dependent promoters. σ^70^ enrichment, as determined by ChIP-qPCR, at FlhDC-dependent promoters and *rpoE* (non-FlhDC promoter) in motile MG1655 and Δ*flhD* (n = 4). σ^70^ enrichment in motile MG1655 was compared to enrichment in Δ*flhD* using two-sample T tests: * *p*<0.05, ** *p*<0.01 (one-tailed).

To provide further insight into the mechanism of regulation at FlhDC-dependent promoters, ChIP-qPCR was used to evaluate σ^70^ occupancy at FlhDC-dependent promoters in wild-type and *flhD* deletion strains (Figure 4). For all positively regulated promoters tested, σ^70^ occupancy was detected in the wild-type strain; however, σ^70^ occupancy was completely undetectable in the Δ*flhD* strain, except at the *yciK*/*sohB* promoter. At that promoter, deletion of *flhD* significantly reduced, but did not eliminate, σ^70^ occupancy, consistent with FlhDC-dependent recruitment to the *yciK* promoter and FlhDC-independent recruitment to the adjacent *sohB* promoter. Our data suggest that FlhDC binding is absolutely required for σ^70^:RNAP recruitment to FlhDC-dependent promoters. This is consistent with reports that these promoters have poor matches to the consensus −10 and −35 hexamers [1].

### Genome-wide binding of FliA

**Figure 5:**
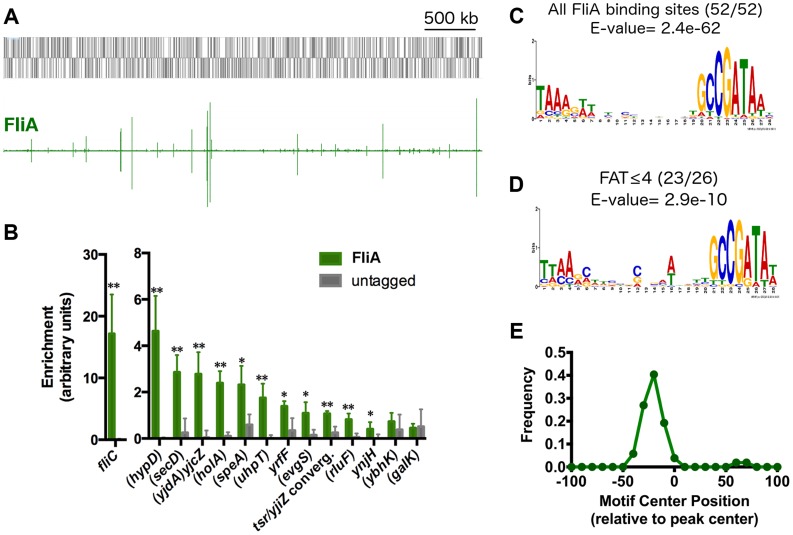
Genome-wide binding of FliA. (A) Genome-wide binding of FliA (green) determined by ChIP-seq. Gray boxes represent gene in the MG1655 genome. (B) ChIP-qPCR enrichment at one well-known (*fliC*) and 13 novel FliA binding sites (n = 3, * *p*<0.05, ** *p*<0.01). Gene names in parentheses indicate that the binding site occurs within that gene. (C) FliA motif derived in this study (all binding sites). (D) Motif derived from binding sites with FAT scores ≤4. Motifs in C and D were generated using MEME [52]. (E) Distribution of motifs relative to ChIP-seq peak centers. Motifs cluster ∼25 nt upstream of the peak center, relative to the orientation of the motif.

To begin assessing the next level of the transcriptional hierarchy, ChIP-seq was used to determine the genome-wide binding of the flagellar σ-factor FliA (Figure 5A). Following a stringent peak-calling analysis, 52 regions were identified that did not overlap with regions enriched in untagged controls (Table 2). These 52 FliA binding sites include 7 canonical flagellar Class 3 promoters and one upstream of the *fliLMNOPQR* operon. FliA binding sites were also identified upstream of five other flagellar-related genes previously shown or predicted to be FliA-dependent: *aer*, *ycgR*, *yhjH*, *trg*, and *tsr*. Additionally, FliA binding sites were identified at 4 other previously reported FliA-dependent promoters of non-flagellar genes: *modA* [30], *ves* [20], *ynjH* [30], and *flxA* [31]. The remaining 35 FliA binding sites represent novel FliA promoters for which no previous experimental evidence can be found, although a small fraction have been predicted by various bioinformatic approaches [51]. Strikingly, 28 of these novel binding sites are inside genes, with only 4 of these intragenic binding sites <300 bp upstream of a start codon for an appropriately oriented annotated gene.

**Table 2:** FliA promoters and expression of associated genes.

					Normalized gene expression^1^	
Peak Center^2^^,^ 3	FAT^4^ Score	Motif Center^2^^,^ 3	Motif	Gene/Operon^5^	Motile MG1655	Δ*fliA* 6
265447	24	265472	GATGAATGCGCTGTGTATTGCCGATAAC	*(yafY)ykfB*	(93)118	(30*)42*
426893	5	426874	AAGGGAATTGCCGTGTTAAACCGTTATC	*(secD)*	183	170
669828	10	669799	TAAAGATTTCATATCAACCGTCGATAAA	*(holA)*	190	170
788978	2	789005	TAAAGTTCGCGTGATGGCAGCCGATTAT	*(galK)*	232	224
794226	32	794199	TCAACTTCCTGCTTTTCCTGCCGATATT	*modABC*	182	61*
815234	4	815204	TCAACTCACGCCCCAGATTGCCGATATA	*(ybhK)*	46	46
816109	2	816141	TAGAGTGTTTAGTGGTTATGCCGATACT	*ybhK*	46	46
946162	2	946134	TATGCTAATGCAGAATTTCTCCGATAAT	*(ycaD)ycaM*	(7)7	(4)7
1030917	1	1030887	TCTGGAACCCTTTCGGGTCGCCGGTTTT	*(serT)hyaA*	(274)0	(154)0
*1049918*	2	*1049983*	TAAGGAAATTGTTACGAAAGCTATTAAT	*insB-4/cspH* converg.	-	-
1129384	48	1129409	TAAAGATTACCCGTCCCTTGCCGATAAA	*flgMN*	451	318
1137565	47	1137556	TCAAGTCCGGCGGGTCGCTGCCGATAAT	*flgKL*	318	138
1241331	2	1241357	TAAGTAAAACGCTGTCTCTGCCGCTAAT	*cvrA*	43	28
1243779	67	1243800	TTAAGTTTTGTTAACTGTGACCGATAAA	*ycgR*	104	0*
1490461	23	1490443	TAAGTAATTACCGTCAAGTGCCGATGAC	*trg*	108	4*
1512636	3	1512671	GCTGGGAATAAACCATATTGCCGATAAA	*(ydcU)*	7	10
1644411	16	1644394	TAAAGATTTTTTTGTGCATGCCGATAGT	*flxA*	280	2*
1676085	1	1676104	TAATATTTTGCGAGTTCACGCCGAAATA	*pntA*	140	169
1823008	44	1823029	AACGTAAATCACCCGAGTTGCCGATAAC	*ves*	18	0
1840188	1	1840186	TAACGTTATTGTCTCTGCTACTGATAAC	*ynjH*	29	2*
1970737	57	1970761	TAAAGTTTCCCCCCTCCTTGCCGATAAC	*tar-tap-cheRBYZ*	251	0*
1975314	101	1975347	TAAACTTTCCCAGAATCCTGCCGATATT	*(flhC)motAB-cheAW*	(320)302	(107*)1*
1979379	2	1979356	TGAAATTGCACCAGATCGAGCCGATAAT	*(otsA)*	26	27
1999845	91	1999853	TGCAGAAACGGATAATCATGCCGATAAC	*fliAYZ*	1257	15*
2001692	110	2001721	TAAAGGTTGTTTTACGACAGACGATAAC	*fliC*	2319	11*
2001856	43	2001841	TAAACTTTGCGCAATTCAGACCGATAAC	*fliDST*	633	356
2017629	18	2017601	TCAAGACGCAGGATAATTAGCCGATAAG	*fliLMNOPQR*	989	980
2232326	3	2232358	TAACAAAACGCTGTAAGCGGCCGATATC	*(preT)*	49	47
2484776	2	2484747	TCAACTTCAACCACAATGGGTCGATATC	*(evgS)*	23	33
2683494	2	2683512	AAAGCGTGAAATGAACATTGCCGATTAT	*(glyA)*	375	415
2850743	51	2850785	TAAAATTATAGGCGTCGGTGCCGATAAC	*(hypD)*	6	7
2860228	2	2860201	TAAGGATCTTGGTCTGGTTGCCGATACA	*(ygbJ)ygbK*	(12)13	(10)10
3082647	11	3082628	TAAAGATGCCGGAAGAGTAGCCGATATG	*(speA)*	133	193
3101896	3	3101871	GGCGCAACGGCAGATTGCTGCCGATAAC	*(mutY)yggX*	(128)369	(104)370
3217148	21	3217162	TAAAGATAACCGCAGCGGGGCCGACATA	*aer*	225	12*
3246148	2	3246175	AAAGCGACCAATTAACAGCGCCGATAAA	*(yqjA)*	140	130
3339939	2	3339902	TAAACTTCTGCTGCGCGTAAACGATATT	*(kdsD)kdsC*	(158)228	(155)241
3524439	9	3524407	CAAGTTAAACTCCACGCTTGCCGATAGC	*yrfF*	62	53
3677244	67	3677273	TAAAGTTCTGCCCTTACGCGCCGATAAT	*yhjH*	511	2*
*3706702*	1	*3706643*	TCCTCTATCACCGACCAAATTCGAAAAG	*(proK)*	62	44
3844022	11	3844039	TAAAAAAGCGATTGGCGCTGCCGATGGT	*(uhpT)*	25	1
3846459	1	3846440	AAAACAGGGTCGCTAACAGGCCGATATC	*(uhpC)*	9	8
4016533	1	4016575	GAGAGTTTTTTCATTGCCTGCCGATAAT	*(rmuC)*	60	78
4119023	1	4118999	TACAGATTTTGTCGATTTCGTCGATAAA	*(hslU)*	1085	1337
4131349	3	4131304	TAAACAGGCGAAGAAATTTGCCGATATG	*(metF)*	6	3
4162805	3	4162795	TGAAGGCGCAGCACGCAGTGACGATAAC	*(btuB)*	72	92
4228823	6	4228793	GAAAGAGTATCTGGTGACGGTCGATAAA	*(rluF)*	73	73
4327161	19	4327146	TAAAGTTCTGGCAGAGCAGGTCGATGAA	*(yjdA)yjcZ*	(111)52	(11*)1*
4564123	1	4564096	GAATAAACTGCAGATCTTTGCCGATATT	*(yjiN)*	17	17
4589660	94	4589638	TAAAGTTTTTCCTTTCCAGGCCGAAAAT	*tsr*	630	9*
4591362	3	4591380	AAAGATTAATCTCCTTATGCCCGATAAC	*tsr/yjiZ* convergent	-	-
4621181	1	4621145	TACAGCCCCCGCCATCCATGCCGATAAC	*(lplA)*	30	31

1 Normalized gene expression values generated by Rockhopper. Expression values are for first gene in operon. Values in parentheses correspond with gene name in parentheses.

2 Peak centers and motif centers refer to genome coordinates relative to NC_000913.2.

3 Peak centers and motif centers in italics are likely false positives, based on the location of the motif relative to the peak center.

4 Fold Above Threshold (FAT).

5 Gene(s) adjacent to binding site. Parentheses indicate an intragenic binding site, while those that are underlined are in the sense orientation and those not underlined are in the antisense orientation.

6 Asterisks indicate significant differential expression (as defined in Methods) between motile MG1655 and Δ*fliA*.

A subset of the putative FliA binding sites identified by ChIP-seq was also tested using ChIP-qPCR. Of the 13 novel FliA binding sites tested, 11 showed significant enrichment in these targeted experiments (Figure 5B). The 2 novel sites that could not be validated had ChIP-seq “fold above threshold” (FAT) scores of 4 or lower. Twenty-two other peaks had similarly low peak scores (≤4) but were not tested by ChIP-qPCR. Sequences surrounding each of the 52 FliA binding sites were extracted and examined for a motif using MEME [52]. A highly significant motif (E-value = 2.4e^−62^) was identified in all 52 binding regions and matches previously reported FliA promoter motifs well (Figure 5C, Table 2). This MEME analysis identified motifs associated with all low-scoring sites, including the two that could not be validated by ChIP-qPCR. To further assess whether low-scoring sites, as a group, show evidence of sequence-specific FliA binding, motifs were identified separately for high-scoring (FAT>4, n = 26) and low-scoring (FAT≤4, n = 26) peaks (Figure 5D). The two groups yielded very similar motifs, both with highly significant q-values (E-value_high_ = 5.8e^−37^, E-value_low_ = 2.9e^−10^). This strongly suggests that a majority of low-scoring ChIP-seq peaks represent genuine sequence-specific FliA binding events.

Not only were motifs identified for all ChIP-seq peaks, but these motifs were also localized in a striking pattern relative to the ChIP-seq peaks (Figure 5E). For most ChIP-seq experiments, one would expect motifs to be enriched at peak centers [53]. However, FliA motifs were clustered with a median position of 25 nt upstream of the peak center (relative to the direction of the motif). This is consistent with the FliA binding to the −10 and −35 hexamer motif while in the context of RNAP holoenzyme [54]. This localization of FliA motifs strongly suggests that FliA only binds in the context of RNAP holoenzyme. Furthermore, this analysis allowed us to identify two weak, non-canonical, putative FliA binding sites (*insB-4/cspH* convergent intergenic region and inside *proK*), that have unusually localized motifs, suggesting that they might not be genuine FliA promoters.

While this manuscript was in preparation, FliA ChIP-chip data was published by another group [55]. Our analysis of this study indicates many false positives and false negatives, and the resolution is far lower than that of our data (Figure S4).

### Direct regulation by FliA

**Figure 6:**
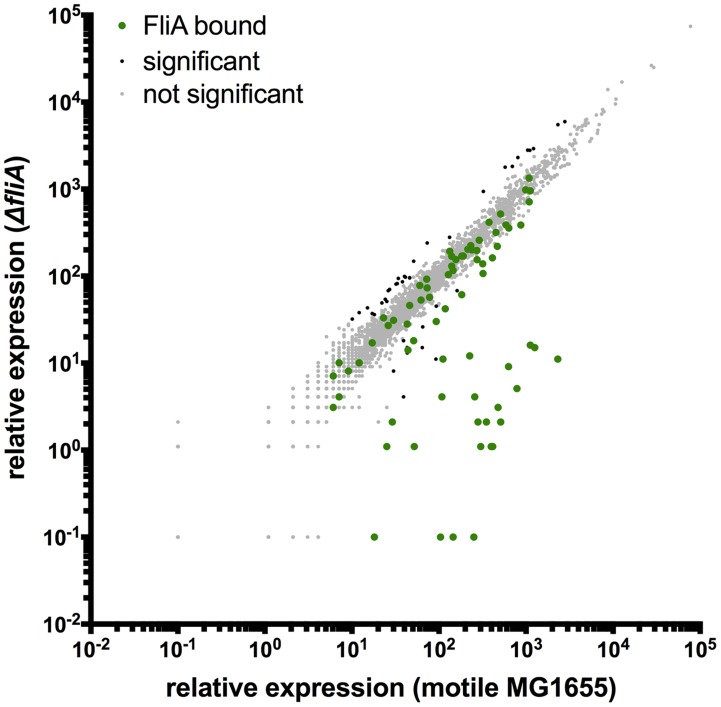
Genome-wide FliA-dependent gene expression. Relative expression of all genes in motile MG1655 versus relative expression in Δ*fliA*. Gene expression values represent normalized expression values calculated by Rockhopper. Green dots represent genes associated with FliA binding. Genes not associated with binding are color-coded according to whether they are significantly regulated (Rockhopper, q-value≤0.01) and changed at least 2-fold (black) or not (grey).

To detect FliA-dependent changes in gene expression, RNA-seq was performed in a Δ*fliA* strain and compared to the isogenic motile wild-type. Analysis of differential gene expression was performed with Rockhopper [36]. Overall, 68 genes were differentially expressed between the motile wild-type and a Δ*fliA* strain (Figure 6). Most of the strongly regulated genes are associated with FliA binding sites identified by ChIP-seq (Figure 6, green points), indicating direct regulation.

**Figure 7:**
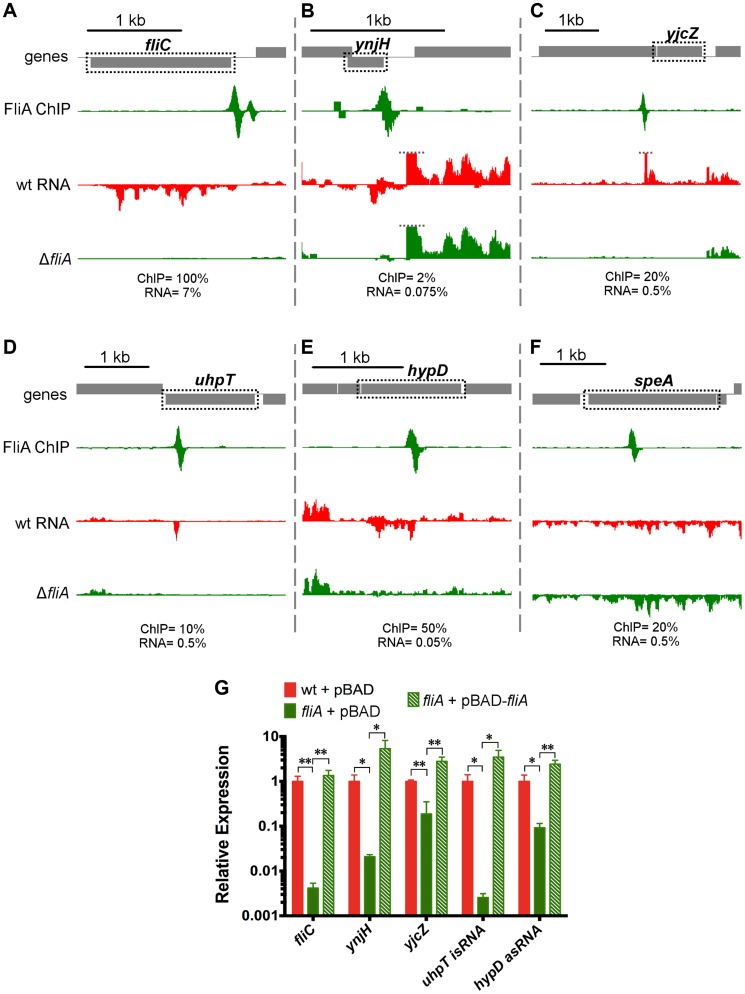
FliA binding and regulation at known and novel targets. (A–F) Mapped reads from ChIP-seq and RNA-seq experiments. Genes and operons of interest are boxed (dotted black line) and labelled. Within each panel, both lanes of ChIP-seq data are scaled equivalently, and all three lanes of RNA-seq are scaled equivalently. Relative scales are indicated below each panel. Dotted gray lines indicate that mapped reads exceed the scale shown. (G) Gene expression, relative to *mreB*, measured by RT-PCR. Gene names are indicated on the x-axis and strain are indicated in the legend (n = 4–7). Statistical comparisons of were performed using two-sample T tests between the indicated groups: * *p*<0.05, ** *p*<0.01 (two-tailed).

Of the 52 FliA binding sites identified by ChIP-seq, 14 were associated with significant gene expression changes under these conditions (Table 2, Figure 7). These included some, but not all, Class 3 promoters, and 8 other previously reported FliA promoters. Interestingly, three of the intragenic FliA binding sites were also associated with significant regulation of surrounding genes. The promoters inside *flhC*, *yjdA*, and *yafY* drive transcription of the downstream genes (*motA*, *yjcZ*, and *ykfB*, respectively; Figure 7C, Table 2).

In addition to using Rockhopper to analyze differential gene expression, we visually inspected mapped RNA-seq data to find FliA-dependent transcripts that might otherwise have been overlooked. We identified an unusual transcript associated with the novel FliA binding site inside *uhpT*. Unlike the previously discussed examples, this promoter drives transcription of a purely intragenic RNA. RNA-seq detects a small (∼50 nt) RNA encoded in the same orientation as the gene (Figure 7D). Visual examination of the RNA-seq data also led to the discovery of an antisense orientation noncoding RNA (asRNA) associated with the novel intragenic FliA-promoter inside *hypD* (Figure 7E). This asRNA is contained entirely within the *hypD* gene and overlaps ∼500 nt of the 5′ end of the *hypD* open reading frame (ORF). The *hypD* antisense RNA is too weakly expressed to be detected by Rockhopper, despite the program's ability to identify novel antisense RNAs. The remaining novel FliA binding sites were not associated with detectable FliA-dependent changes in gene expression under these conditions (eg. *speA*, Figure 7F).

A variety of canonical and novel examples of FliA-dependent regulation were further validated using qRT-PCR (Figure 7G, Table S2). In all cases, including the two novel non-coding RNAs, the regulation identified in the RNA-seq experiment was confirmed in these targeted experiments. Additionally, overexpression of *fliA* in the Δ*fliA* strain resulted in higher than wild-type levels of expression of all FliA-dependent transcripts tested.

### Dual regulation of complex promoters and overlapping operons

**Figure 8:**
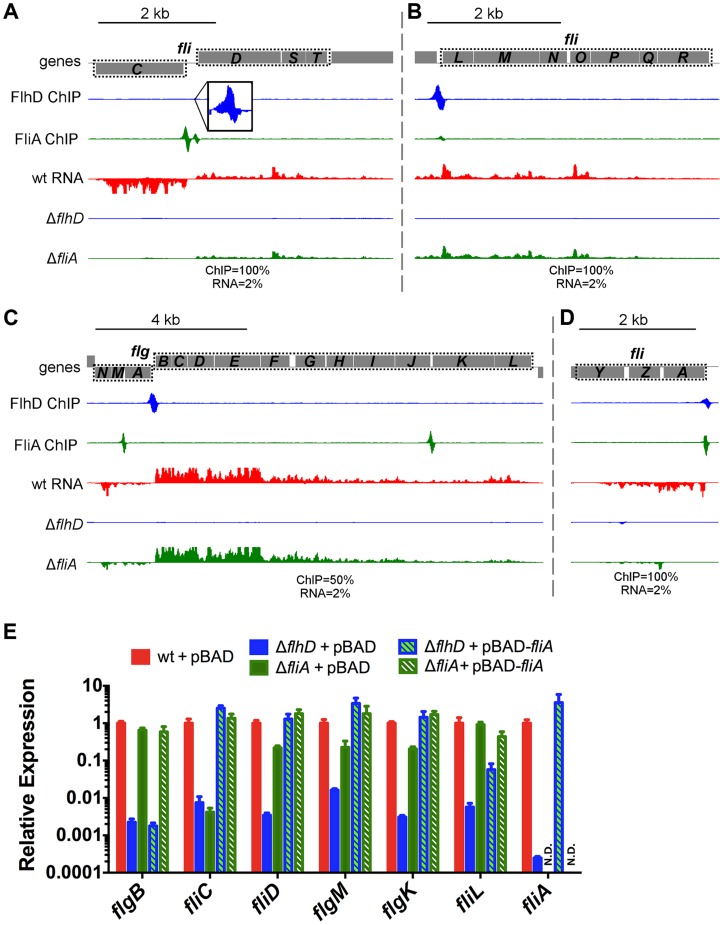
FlhDC and FliA binding and regulation at dual regulated targets. (A–D) Mapped reads from ChIP-seq and RNA-seq experiments. Genes and operons of interest are boxed (dotted black line) and labelled. Within each panel, both lanes of ChIP-seq data are scaled equivalently, and all three lanes of RNA-seq are scaled equivalently. Relative scales are indicated below each panel. Dotted gray lines indicate that mapped reads exceed the scale shown. (E) Gene expression, relative to *mreB*, measured by RT-PCR. Gene names are indicated on the x-axis and strain are indicated in the legend (n = 4–7). Due to the high number of potential comparisons, statistical analysis is presented in Table S2.

Complex promoters and overlapping operons were first evaluated using our ChIP-seq and RNA-seq data (Figure 8). The previously described (*fliAZY* [22] and *fliLMNOPQR* [22]) or predicted (*fliDST* [24]) complex promoters are supported by our findings (Figure 8A,B,D). The previously described overlapping *flgAMN/flgMN* operons are also supported by our data (Figure 8C). Finally, our data confirm that *flgKL* can be transcribed as part of the upstream FlhDC-dependent *flgBCDEFGHIJ* operon, in addition to being transcribed from its own Class 3 promoter (Figure 8C, Table 2). While these operons have been previously described in *Salmonella* [56], [57], this is the first experimental demonstration of the overlapping operons in *E. coli*. The novel *flgBCDEFGHIJKL* operon was further confirmed by using RT-PCR to amplify across the *flgJ-flgK* boundary (Figure S5A).

In addition to confirming dual regulation of *fliDST* and discovering that *flgKL* can be transcribed as part of the upstream operon, the RNA-seq data from this study reveal a surprising pattern for all dual regulated flagellar genes. In the investigated conditions and growth phase, deletion of FliA has little or no effect on the expression levels of dual regulated genes (Figure 8A–D). This suggests that, while FliA occupancy is detected at all dual promoters by ChIP-seq, most transcription is coming from the FlhDC-dependent σ^70^ promoters under our conditions.

We used qRT-PCR to further investigate the regulatory input of FlhDC and FliA for dual regulated genes (Figure 8E). As seen in the RNA-seq experiments, deletion of *fliA* had a much less dramatic effect on gene expression compared to deletion of *flhD*. Overexpression of *fliA* in the Δ*flhD* strain significantly increased RNA levels of all dual targets (compared to Δ*flhD*) showing that the FliA promoters are capable of transcribing the target genes. However, this effect was smaller for *fliL* than for any other dual regulated gene. Furthermore, overexpression of *fliA* in the Δ*fliA* strain (hashed green bar) reduced *fliL* expression by 2.10-fold compared to the empty vector control (solid green bar; t-test, *p*-value 0.02), suggesting a possible repressive interaction (Figure 8E).

As dual regulation has been suggested for all Class 2 targets [23], we performed similar qRT-PCR experiments for these genes. Overexpression of *fliA* in the Δ*flhD* strain was not able to significantly increase the expression of any other Class 2 target tested (Figure S6). While not statistically significant, *flhB* expression does increase with *fliA* overexpression (Figure S6). Since *flhB* is downstream of the FliA-dependent *meche* operon, it is possible that the *flhBAE* operon is dual regulated and can be transcribed as part of the upstream operon. However, there is no evidence of co-transcription from the RNA-seq or RT-PCR targeting the *cheZ-flhB* intergenic region (Figure S5B) so we conclude that this read-through is likely an artifact of extreme FliA overexpression. It should also be noted that no FliA ChIP-seq signal was detected in the vicinity of the *flhBAE*, *fliE*, or *fliFGHIJK* operons, nor were any of these Class 2 genes differentially expressed in RNA-seq experiments (motile wild-type and Δ*fliA* strains). Therefore, with the exception of the dual targets already discussed, our data do not support widespread dual regulation of Class 2 genes.

### Motility assessment of novel FlhDC and FliA targets

To determine if novel FlhDC and/or FliA targets were required for motility, knockouts were generated for *ampH*, *ymdA*, *sbmA*, *gntR*, *ykfB*, *yjcZ*, *ynjH*, *hypD*, and *uhpT*. Motility on semi-solid agar was determined for each deletion strain. None of the deletions resulted in a substantial change in motility (Figure S7), suggesting that these genes do not directly contribute to motility under the conditions tested.

## Discussion

### FlhDC regulon

To our knowledge, this study provides the first look at genome-wide *in vivo* binding of FlhD and FlhC. We have redefined the direct FlhDC regulon by identifying new targets and showing that many previously reported non-flagellar targets are incorrect or not bound under these conditions (Table 1, Figure S2). Our data support a surprisingly limited direct FlhDC regulon and show no evidence of either protein binding DNA independently. Under the conditions tested, FlhDC binds 11 loci and directly regulates 11 transcriptional units. However, it is important to note that some binding sites are associated with regulation of two divergent operons while others are not associated with any detectable regulation. In addition to the classical flagellar Class 2 binding sites, our study detects previously reported binding sites upstream of *yecR* and *fliDST*, and 4 novel FlhDC binding sites. Two of these novel binding sites were associated with detectable FlhDC-dependent regulation of adjacent genes (*ymdA* and *yciK*), while the remaining two were not (*gntR* and *ampH/sbmA*). It is likely that the two latter sites are functional, perhaps under different conditions, because the probability of two spurious sites occurring in intergenic regions is very small (only ∼11% of the genome is intergenic sequence). None of the genes surrounding the 4 novel binding sites have an obvious functional connection to flagellar synthesis, and deletion of these genes had no detectable effect on motility (Figure S7). The RNA-seq data provided little evidence of an extensive indirect FlhDC regulon. While 183 genes were differentially regulated but not associated with FlhDC or FliA binding, the magnitude of the indirect regulation is significantly smaller than for direct targets (Figure 2; Krustal-Wallis, *p*<0.0001). FliZ is the only transcription factor regulated directly by either FlhDC or FliA, and we do not see regulation of described FliZ targets [58]. Therefore, we doubt that FliZ-dependent regulation substantially contributes to the indirect FlhDC-dependent regulation detected in this study. Instead, it is likely that the indirectly regulated genes do not represent an additional level of the FlhDC transcriptional network but result from physiological and metabolic changes associated with flagellar motility.

Despite having a degenerate consensus motif, FlhDC appears to bind DNA highly specifically- at only 11 sites throughout the genome. The presence of a motif alone is insufficient for binding *in vivo*, even at sites that are bound *in vitro* (Figure S2). We propose that as-yet unidentified factors such as DNA conformation or competition with nucleoid-associated proteins play a role in the surprising specificity of FlhDC.

Although the presence of a motif and *in vitro* binding does not always predict *in vivo* FlhDC behavior, a striking pattern emerges for flagellar Class 2 sites. There is a perfect correlation between reported *in vitro* FlhDC affinities [54], expression kinetics [59], and *in vivo* FlhDC occupancy (this study). Coupled with the σ^7^°ChIP data presented here (Figure 4), this suggests a simple mechanism of transcriptional activation: σ^70^-RNAP is unable to bind these promoters in the absence of FlhDC, but as its concentration increases, FlhDC binds DNA and recruits σ^70^-RNAP to promoters in the order of relative FlhDC binding affinity. This model of affinity-based temporal ordering has been commonly predicted in transcriptional networks [60]. Furthermore, this suggests that for transcription factors with a similar recruitment-based mechanism of action, relative *in vivo* occupancy derived from ChIP-seq can be used to predict temporal expression patterns.

### FliA regulon

 By coupling ChIP-seq and RNA-seq, we have comprehensively identified FliA binding sites and matched some of these with FliA-dependent transcripts. We identified 52 FliA binding sites, 35 of which are novel. Furthermore, we have demonstrated that existing computational approaches [30], [32], [33], [51] are poor predictors of *in vivo* FliA binding. Our data confirm all the better-characterized members of the FliA regulon while discounting most promoters for which only bioinformatic predictions exist (Table S3). It should be noted that our findings largely agree with a previous microarray expression study of the FliA regulon [20]. However, we have greatly expanded the regulon and identified precise promoter positions, many of which are different from those predicted by Zhao *et al.* based on their ORF-based expression data [20].

Under the conditions used in our study, 14 out of 52 FliA binding sites were associated with significant changes in the RNA levels of one or more surrounding genes (wild-type vs. Δ*fliA*). This suggests, as will be discussed below, that a large number of promoter sequences bind FliA but are relatively inactive. Similar to FlhDC, the RNA-seq data provide little evidence for an extensive indirect FliA regulon. Only 40 genes were differentially regulated but not associated with FliA binding. The magnitude of indirect regulation was dramatically smaller than direct regulation (Figure 6; Mann-Whitney, p<0.0001). As with FlhDC, it seems likely that indirect regulation is due to secondary physiological and metabolic changes associated with flagellar motility. Consistent with this idea, 29 of the 40 indirectly regulated genes are also indirectly regulated by FlhDC.

### Non-canonical FliA binding sites

The most striking finding from our FliA ChIP-seq experiments is that more than half of FliA binding sites are inside genes. While intragenic binding has been reported for other σ-factors and for many transcription factors, the proportion of intragenic FliA sites is remarkable. Similar to our findings, 40% of the sites bound by the *Mycobacterium tuberculosis* σ-factor, SigF, are intragenic. Some of these sites are associated with SigF-dependent transcription in the antisense orientation relative to the overlapping gene [61]. RpoH (σ^32^) has been reported to bind many intragenic sites, but these sites only account for ∼25% of the total sites bound (22 out of 87, [62]). Lastly, a recent study reported that σ^70^ can bind and initiate transcription at a large number of intragenic sites, but that this phenomenon is repressed at many locations by the nucleoid-associated protein H-NS [63]. Combined with our FliA results, it is clearly important to take intragenic transcription initiation into account when analyzing genome-wide data.

Of the 30 intragenic FliA sites identified in our study, only 5 are associated with detectable changes in RNA levels. Three intragenic promoters appear to drive transcription of canonical mRNAs for the downstream gene(s). Two of these promoters have been accurately predicted before (inside *flhC* driving *motAB-cheAW* [32], [64], and inside *yafY* driving *ykfB* [20]), but one is novel (inside *yjdA* driving *yjcZ*). A FliA promoter upstream of *yjdA* has been incorrectly predicted, based on expression data [20]. These three sites function as canonical promoters and are likely inside genes simply due to the spatial constraints of a small genome. Additionally, two novel intragenic FliA binding sites are associated with detectable small, intragenic RNAs. The FliA promoter inside *uhpT* transcribes a small RNA overlapping the 3′ end of the ORF in the sense orientation (Figure 7D), while the FliA promoter inside *hypD* drives transcription of an antisense RNA (Figure 7E). A FliA promoter upstream of *uhpT* has been predicted based on expression data [20], but our data clearly show that the FliA-dependent transcript is not an mRNA. While the FliA promoters inside *uhpT* and *hypD* are associated with detectable transcription, the functions of the corresponding RNAs are not known. Neither intragenic RNA shows evidence of regulating the overlapping mRNA. It is possible that one or both of these RNAs regulate *trans*-encoded targets.

Most intragenic FliA binding sites are not associated with detectable transcripts. Furthermore, most of these putative promoters are greater than 300 nucleotides upstream of the start codon to an adjacent gene making it improbable that they drive transcription of canonical mRNAs. More likely, these promoters, if active, transcribe non-coding RNAs. However, since no changes in local RNA levels were detected flanking these binding sites, it remains unclear whether these FliA binding sites represent functional FliA-dependent promoters. Many studies have reported widespread spurious transcription, most of which is rapidly terminated and produces highly unstable transcripts [65]. Therefore, these promoters may be active but the resulting RNAs are too unstable to detect by standard RNA-seq methods. Three of these intragenic promoters were previously predicted in *E. coli* and two of the three, those inside *galK* and *speA*, showed FliA-dependent activity when cloned upstream of the chloramphenicol acetyltransferase reporter gene [64]. This supports the hypothesis that at least some of the intragenic FliA binding sites are functional promoters, even if no transcripts were detectable in our study. Alternatively, these binding sites could represent promoter-like sequences where FliA∶RNAP can bind but cannot initiate transcription under the conditions tested, or potentially, ever. Regardless of their transcriptional activity, intragenic FliA sites could serve to alter the available pool of FliA, indirectly affecting transcription from canonical promoters, as has been proposed for some transcription factors [66], [67].

### Network topology and the consequences of dual regulation

**Figure 9:**
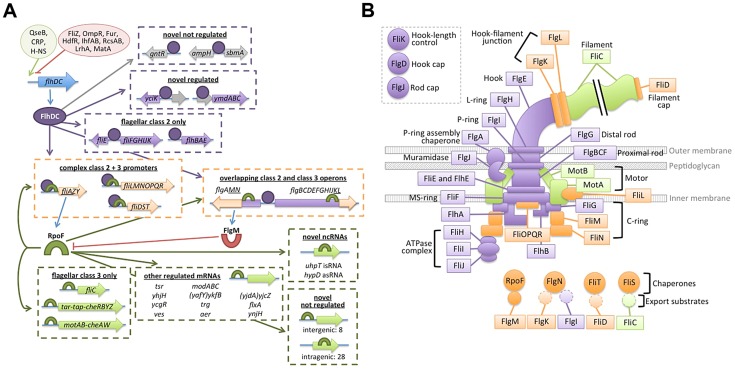
Updated flagellar transciption network and localization of dual-regulated targets. (A) The transcription network map has been updated to accurately depict dual-regulated targets and to incorporated novel regulon members. Ovals at top left represent positive (green) and negative (red) regulation of *flhDC* transcription. Blue arrows indicate translation of gene products. Purple ovals represent FlhDC and purple arrows indicate FlhDC regulatory interactions. Green cresents represent FliA and green arrows represent FliA regulatory interactions. Dotted arrows indicate potential regulation of genes associated with binding sites but without detectable regulation in this study. Target genes/operons are boxed according to their regulatory input: FlhDC only (green), FliA only (green), and dual FlhDC and FliA (orange). (B) Localization and function of flagellar gene products, color-coded by regulatory input as described for panel B. Gene products not physically associated with the flagellum are omitted.

Over the last 15 years there has been a growing interest in modeling cellular processes as networks [60]. Several frequently occurring network motifs have been identified, with feed-forward loops (FFLs) being one of the most common. In FFLs, one regulator regulates another regulator, and both regulate a common target gene (or genes) [68]. A quantitative computational model of the flagellar network has been constructed [23] and is considered a seminal work in the field of network modeling [69]. A key aspect of the existing flagellar network model is that all Class 2 genes are modeled as FFLs with dual FlhDC/FliA input. However, our data clearly indicate that three Class 2 operons (10 genes) are not dual regulated (Figure S6, Figure 9A). This implies that the true behavior of the transcriptional network cannot be accurately predicted by the existing model.

In addition to clarifying the overall topology of the network, our genome-wide and targeted experiments have revealed interesting information about the regulatory inputs for specific dual targets. One striking pattern that emerges is that, while FliA binding is readily detected at all dual targets, RNA levels detected by RNA-seq and qRT-PCR only change moderately between wild-type and Δ*fliA*. In most cases, expression of dual regulated genes is affected less than 4-fold by *fliA* deletion, but more than 100-fold by *flhDC* deletion (Figure 8). This suggests FliA-dependent transcription is contributing very little to the overall abundance of dual regulated transcripts under these conditions, despite FliA being present at the associated promoters. Despite the seemingly low level of activity of these FliA promoters, when *fliA* is overexpressed in a Δ*flhD* strain, expression of *fliD*, *flgM*, and *flgK* returns to wild-type or higher levels. This demonstrates that these promoters do have the capacity to be very active, but it remains to be seen if FliA levels and activity are ever high enough to trigger high-level promoter activity in wild-type cells. Interestingly, *fliA* overexpression results in lower *fliL* expression in both Δ*flhD* and Δ*fliA* strains compared to wild-type (Figure 8E). Since the FliA promoter is downstream of the FlhDC-dependent σ^70^ promoter, we speculate that high FliA∶RNAP occupancy yields little FliA-dependent transcription and actually competes with σ^70^:RNAP at the level of DNA binding to repress transcription from the upstream promoter. Overall, our data suggest that flagellar genes likely have diverse temporal expression patterns due to the diversity of regulatory inputs at each promoter (Figure 9A).

Now that we have systematically identified dual-regulated genes, it is tempting to surmise why certain genes are under dual control, and others are not. The correlation between the gene expression class (Class 2 or 3) and the developmental stage at which the resulting proteins are utilized has been described (reviewed [3]). However, dual-regulated gene products show less of a pattern in localization or correlation with a specific phase of assembly (Figure 9B). In *Salmonella*, the Class 3 promoters of *fliDST* and *flgKL* have been shown to be required for swarming and for rapid repair of sheared flagella [57]. The authors hypothesized that these genes utilize their Class 2 promoters during *de novo* flagellar assembly but are transcribed from the Class 3 promoters, independent of FlhDC activity, to repair flagella broken during swarming. Wozniak *et al.* also suggest that *flgMN* is dual regulated so that FlgM can fine-tune FliA activity throughout flagellar assembly and so that FlgN can assist in FlgK folding and transport during flagellar repair [57]. The final dual-regulated operon identified in our study, *fliLMNOPQR*, encodes components of the C-ring and secretion apparatus that localize to the proximal portion of the flagella (Figure 9B). Our results suggest that high levels of FliA may repress this operon (Figure 8E) but it is unclear why these components would be specifically targeted by negative feedback. Alternatively, FliA may positively regulate the *fliL* operon at some stage of flagellar development but it is equally unclear why this would be required. The specific roles of the Class 2 and Class 3 promoters of dual-regulated targets should be explored further to determine the physiological importance of the complex temporal gene expression patterns resulting from dual regulation.

### Conclusions

We have identified many new targets of FlhDC and FliA, including many non-canonical FliA binding sites. Additionally we have shed new light on the complex topology of the network by systematically defining Class 2, Class 3, and dual-regulated targets. Finally, we have demonstrated that the combined application of ChIP-seq and RNA-seq to related regulators provides sufficient data to build a transcriptional network model from scratch or redefine even the best-characterized networks, such as the flagellar transcription network.

## Materials and Methods

### Strains and plasmids

Bacterial strains used in this work are listed in Table S4. Cells were grown in Lysogeny Broth (LB: 1% NaCl, 1% tryptone, 0.5% yeast extract) or Tryptone broth (TB: 1% tryptone, 0.5% NaCl). Strains harboring plasmids were cultured with the appropriate antibiotic, as listed in Table S4.

Primers used in strain construction are described in Table S5. Epitope-tagged strains (DMF11, DMF14, RPB081) were generated using the FRUIT method of recombineering [70]. All epitope-tagged strains originate from AMD052 (MG1655 Δ*thyA*), which has a poorly motile phenotype. FliA was N-terminally tagged by inserting a 3×FLAG tag after the third amino acid. FlhD and FlhC were tagged by inserting 3×FLAG tags into internal loop regions (Figure S1A). Internal sites were chosen since the termini of both proteins are known or predicted to participate in protein-protein and/or protein-DNA contacts [42], [43]. Following motility selection (see below), the region upstream of *flhDC* and the *flhDC*-coding region were sequenced in each strain (Wadsworth Center Applied Genomic Technologies Core; File S1). Strains DMF62–65 were also generated using FRUIT. In each strain, the *thyA* cassette was inserted (facing away from *flhDC*) at a precise location upstream of *flhDC*. *thyA* insertions were confirmed by PCR and sequencing (Wadsworth Center Applied Genomic Technologies Core).

Deletion strains (Δ*flhD*, Δ*flhC*, and Δ*fliA*) were also generated using recombineering. First, DMF35 was generated from AMD052 by motility selection, as described below. With DMF35 as the common parent strain, FRUIT was used to generate scarless deletions of *flhD* (DMF38) and *fliA* (DMF40). The *flhC* deletion strain (DMF58) was generated by amplifying the *flhC*::kan allele from the Keio collection [71], electroporating it into DMF35, and then removing the kan cassette by expressing FLP recombinase from pCP20 [72]. Following tag insertion or gene deletion, *thyA* was reintroduced at its native locus and strains were cured of all plasmids. Strains DMF50–57 were also generated from AMD052, using FRUIT to replace the genes of interest with the *thyA* gene. Note that these strains lack *thyA* at its native locus, but have a *thyA* ^+^ phenotype. Overexpression plasmids were generated by cloning the ORF of interest into pBAD24 [73] cut with *NheI* and *SphI* (NEB) using the In-Fusion method (Clontech). All deletion strains and plasmids were verified by PCR and sequencing (Wadsworth Center Applied Genomic Technologies Core).

### Motility selection to generate motile isolates

Our lab isolate of MG1655, and the related strain AMD052, displayed a poorly motile phenotype. PCR using JW3100 and JW5358 demonstrated that these strains lacked an IS element upstream of the *flhDC*, which is required for high-level motility. Epitope tagged strains were generated from AMD052, and were thus initially poorly motile as well. To isolate highly motile derivatives, saturated overnight cultures of strains AMD052 and preliminary epitope-tagged strains were spotted (5 µL) onto soft TB agar (0.3%) and incubated at 30°C. Motile subpopulations began emerging between 20 and 24 hours. We typically observed 1–5 motile subpopulations on each plate. Motile cells were collected from stabs, yielding motile strains DMF35, DMF11, DMF14, and RPB081. IS element insertion was verified by PCR and sequencing with oligonucleotides JW3100 and JW5358 (Wadsworth Center Applied Genomic Technologies Core; File S1).

### Motility assays

Overnight cultures were grown in TB at 30°C. Saturated cultures (5 µL) were spotted onto 155 mm TB soft agar (0.2%) plates and incubated at 30°C. Each plate included DMF36 for reference and 1–4 other strains of interest. Each assay was performed 5 times from independent overnight cultures. Images were taken at hourly intervals from 4–6 hours post-inoculation. Representative images are shown in Figure S1 and S7.

### ChIP-qPCR

 Strains DMF11, DMF14, RPB081, and DMF36 were used for all ChIP experiments. Subcultures were grown in LB at 37°C with aeration to an OD_600_ of 0.5–0.7. Cultures were harvested, crosslinked, sonicated, and immunoprecipitated as previously described [74], with minor modifications. Anti-FLAG (2 µL per IP, M2 monoclonal; Sigma) or anti-σ^70^ (1 µL per IP; Neoclone) antibodies were used for all immunoprecipitations, both for ChIP-qPCR and ChIP-seq. ChIP and input DNA was purified using Zymo PCR Clean and Concentrate kit. Samples were analyzed using qPCR as previously described [74]. Oligonucleotides used to amplify the *bglB* control region and regions of interest are described in Table S6. ChIP-qPCR experiments were utilized 3–7 biological replicates per strain. Complete ChIP-qPCR data is presented in Table S1.

### ChIP-seq library preparation and sequencing

Cultures for ChIP-seq experiments were grown as described for ChIP-qPCR. ChIP-seq libraries were constructed and sequenced as previously described [63] with the exception of one replicate of FlhC-FLAG, which was prepared as described [37]. Antibodies used were the same as those used for ChIP-qPCR. ChIP-seq libraries were constructed and sequenced for 2 biological replicates per strain. Sequencing was performed on an Illumina Hi-Seq instrument (University at Buffalo, SUNY).

### ChIP-seq data analysis

Sequences were aligned to the MG1655 genome (NC_000913.2) using the CLC Genomics Workbench. Mapped reads were piled up and written to a .gff file using a custom Python script and viewed in SignalMap (Nimblegen). All ChIP-seq images presented in this study are captured from SignalMap and manipulated in the image editing software GIMP to highlight baselines (zero reads) and fill gaps in the data resulting from image artifacts.

Almost all ChIP-seq analysis programs have been designed and optimized for eukaryotic ChIP-seq data and, in our experience, do not perform well with bacterial ChIP-seq data. We have generated custom Python scripts to identify peaks in bacterial ChIP-seq data. First, all datasets were normalized to 100 million reads. Pairs of replicate datasets were considered together. For each replicate dataset in the pair, an appropriate threshold was determined. The plus and minus strands were considered separately. For the first replicate, for a given strand, a value *T_1_* was selected as the threshold. For the second replicate, a value *T_2_* was selected as the threshold. Values for *T_1_* and *T_2_* were considered between 1 and 1000. For each combination of values for *T_1_* and *T_2_*, the number of genome positions with values ≥*T_1_* in the first replicate and with values ≥*T_2_* in the second replicate was determined. The false discovery rate was estimated using the null hypothesis that no regions are enriched. The combination of thresholds yielding the highest number of true positive positions, with an estimated false discovery rate of less than 0.01, was selected. Once *T_1_* and T*_2_* were chosen, peak calling was performed as previously described (Supplementary Material of [54]). Briefly, a region was identified as a peak if both replicates showed enrichment above the corresponding thresholds for each strand. For a peak to be called there must be a peak on the plus strand within a threshold distance of a peak on the minus strand, as previously described (Supplementary Material of [54]). To identify regions of artifactual enrichment, peaks identified in tagged strains were compared to those called in a control ChIP-seq experiment using an untagged strain (DMF35). For each factor, the calculated *T* values were adjusted to reflect the total number of reads in control experiment replicates and then applied for peak calling in the controls. Any regions for which a peak was called in the true ChIP-seq experiment and in the untagged control experiment within 50 bp of each other were considered potential artifacts and excluded from further analysis.

### RNA isolation and RNA-seq library preparation

Strains DMF36, DMF38, DMF58, and DMF40 were used for RNA-seq experiments. Subcultures were grown in LB at 37°C with aeration to an OD_600_ of 0.5–0.7. RNA was purified using a modified hot phenol method, as previously described [37]. Following isolation, RNA was treated with DNase (TURBO DNA-free kit; Life Technologies) for 45 minutes at 37°C, followed by phenol extraction and ethanol precipitation. rRNA was removed using the RiboZero kit (Epicentre) and strand-specific DNA libraries were constructed using the ScriptSeq 2.0 kit (Epicentre). Sequencing was performed using an Illumina Hi-Seq instrument (University at Buffalo, SUNY).

### RNA-seq data visualization and differential expression analysis

Sequence reads were mapped and visualized as described above. Differential expression analysis was performed using Rockhopper [36] with default parameters. As suggested by the developers, changes in gene expression were considered statistically significant if *q*-value≤0.01. Genes were required to be regulated at least 2-fold to be considered significantly differentially regulated. Ribosomal RNAs (rRNAs) were excluded from RNA-seq analysis since rRNA was removed during library preparation.

### RNA isolation and qRT-PCR

Strains pDMF9–18 were used for qRT-PCR experiments. Subcultures were grown in LB+100 µg/ml ampicillin at 37°C with aeration to an OD_600_ of 0.4–0.6. Arabinose was added to a final concentration of 0.2% and cultures were incubated at 37°C for an additional 10 minutes. RNA was isolated as follows. 10 ml of culture were harvested by centrifugation, resuspended in 1 ml Trizol reagent (Life Technologies) and incubated at room temperature for 5 min. Samples were centrifuged, supernatants were transferred to new tubes, and 200 µL of chloroform was added. Samples were mixed well, incubated at room temperature for 3 minutes, and centrifuged. The aqueous layers were transferred to new tubes and mixed with 500 µL isopropanol. Samples were mixed well, incubated at room temperature for 10 minutes, and centrifuged to collect precipitated RNA. Pellets were washed once with 75% ethanol and then dried. RNA was resuspended in H_2_O and treated with TURBO DNase (Life Technologies) as described above. RNA was reverse transcribed using SuperScript III reverse transcriptase (Invitrogen) with 100 ng of random hexamer, according to the manufacturer's instructions. A control reaction lacking reverse transcriptase (no RT) was performed for each sample. cDNA and no RT samples were diluted and used as templates for qPCR. qPCR was performed with an ABI 7500 Fast real time PCR machine. Oligonucleotides used to amplify target genes, and the control (*mreB*) are listed in Table S7. Relative expression values were calculated using a modified 2^−ΔΔCt^ method [75]. First target Ct values were normalized to the control (*mreB*) yielding ΔCt values, then 1.9^−ΔCt^ (assuming imperfect PCR efficiency) was calculated for each target in each strain. Finally, expression values in all strains were normalized to the average 1.9^−ΔCt^ value in motile MG1655+pBAD. qRT-PCR experiments utilized 3–4 biological replicates per strain and all qPCR reactions were performed in triplicate.

## Supporting Information

Figure S1 **Strain construction and validation.** (A) Tag locations for internal 3×FLAG-tagging of FlhD and FlhC. Black lines represent unstructured regions, red cylinders represent α-helices, and blue boxes represent β-sheets. Gold stars represent Zn-binding cysteine residues. Insets show amino acid sequence surrounding tag insertion sites. (B) Location, identity, and direction of IS element insertions present in each motility-selected strain. The boxes represent regions that are duplicated during insertion. (C) Soft agar motility of motile MG1655 and strains in which a *thyA* cassette has been inserted in each of the IS element insertion locations described above. (D) Soft agar motility of motile MG1655 and epitope-tagged strains. (E) Soft agar motility of motile MG1655, isogenic deletions, and complemented strains. Note that Δ*flhC+*p*flhC* is non-motile due to disruption of the promoter of the *motAB-cheAW* operon.
(PDF)

Figure S2 **Presence of FlhDC motif and** ***in vitro*** **binding is not predictive of** ***in vivo*** **binding.** Stafford *et al.* [25] predicted FlhDC binding sites based on the consensus motif of characterized binding sites. The sites shown in this figure had good matches to the consensus and demonstrated weak *in vitro* binding. With the exception of *fliD* and *yecR* (not shown), none of the predicted sites showed *in vivo* FlhDC binding in targeted ChIP-qPCR assays (n = 4, * *p*<0.05, ** *p*<0.01).
(PDF)

Figure S3 **Genome-wide expression in Δ** ***flhD*** **and Δ** ***flhC*** **.** Expression of all genes in Δ*flhD* versus Δ*flhC*. Gene expression values represent normalized expression values calculated by Rockhopper.
(PDF)

Figure S4 **Comparison of FliA motifs from this study and Cho** ***et al.*** **** [**55**] **.** Motifs were generated from sequence surrounding binding sites identified only in our study (n = 27) or only in Cho *et al.* (n = 29). (A) FliA binding sites unique to our study yielded a highly significant motif (27/30 sites, E-value = 1.5e-27) similar to that described for FliA. (B) The best-scoring motif for FliA binding regions unique to Cho *et al* is not significantly enriched, and shows no similarity to described FliA motifs (best-scoring motif: 10/29, E-value = 3.5). It should also be noted that Cho *et al* failed to detect some well-characterized FliA promoters such as those upstream of *fliAZY* and *fliC*.
(PDF)

Figure S5 ***flgJK*** **is dual regulated but** ***flgBAE*** **is not.** (A) RT-PCR using an upstream primer within *flgJ* and a downstream primer within *flgK* yielded a band of the expected size in motile MG1655, but not in Δ*flhD*. This confirms that *flgKL* can be transcribed as part of the upstream FlhDC-dependent operon. Lanes labeled “colony” are a colony (genomic DNA) PCR control, “+RT” are RT-PCR, and “−RT” are controls in which no reverse transcriptase was added during cDNA synthesis. (B) RT-PCR using an upstream primer within *cheZ* and a downstream primer within *flhBAE* yielded very little product. Product slightly increased, relative to the −RT control, when *fliA* was overexpressed. This small amount of read-through at very high FliA levels is unlikely to be physiologically relevant.
(PDF)

Figure S6 **All class 2 genes are not transcribed by FliA.** Expression of *flgA*, *flgB*, and *fliF* cannot be rescued by overexpression FliA in Δ*flhD*. Expression of *flhB* is moderately increased in the Δ*flhD*+pBAD-*fliA* strain, potentially due to read-through from the upstream FliA-dependent *tar-tap-cheRBYZ* (see Figure S4).
(PDF)

Figure S7 **Novel FlhDC and FliA targets are not required for motility.** Soft agar motility of motile MG1655 and single gene deletions of FlhDC and FliA target genes. Images are representative of 5 biological replicates per strain.
(PDF)

Table S1 Complete ChIP-qPCR data.
(XLS)

Table S2 Complete qRT-PCR data.
(XLS)

Table S3 Comparison of previously predicted FliA promoters to ChIP-seq binding sites.
(XLS)

Table S4 Strains.
(XLS)

Table S5 Oligonucleotides: strain construction and validation.
(XLS)

Table S6 Oligonucleotides: ChIP-qPCR.
(XLS)

Table S7 Oligonucleotides: qRT-PCR.
(XLS)

File S1 Sequence of *flhDC* locus in motile MG1655 and epitope-tagged strains.
(DOCX)
