# Correction: Comprehensive Mapping of the *Escherichia coli* Flagellar Regulatory Network

**DOI:** 10.1371/journal.pgen.1004807

**Published:** 2014-10-20

**Authors:** The PLOS Genetics Staff

There is an error in the legend for Table 2. Footnote 5 is incorrect. It should read “Gene(s) adjacent to binding site. Parentheses indicate an intragenic binding site, while those that are underlined are in the antisense orientation and those not underlined are in the sense orientation.”

**Table 2:** FliA promoters and expression of associated genes.

					Normalized gene expression^1^	
Peak Center^2^^,^ 3	FAT^4^ Score	Motif Center^2^^,^ 3	Motif	Gene/Operon^5^	Motile MG1655	*ΔfliA* 6
265447	24	265472	GATGAATGCGCTGTGTATTGCCGATAAC	*(yafY)ykfB*	(93)118	(30*)42*
426893	5	426874	AAGGGAATTGCCGTGTTAAACCGTTATC	*(secD)*	183	170
669828	10	669799	TAAAGATTTCATATCAACCGTCGATAAA	*(holA)*	190	170
788978	2	789005	TAAAGTTCGCGTGATGGCAGCCGATTAT	*(galK)*	232	224
794226	32	794199	TCAACTTCCTGCTTTTCCTGCCGATATT	*modABC*	182	61*
815234	4	815204	TCAACTCACGCCCCAGATTGCCGATATA	*(ybhK)*	46	46
816109	2	816141	TAGAGTGTTTAGTGGTTATGCCGATACT	*ybhK*	46	46
946162	2	946134	TATGCTAATGCAGAATTTCTCCGATAAT	*(ycaD)ycaM*	(7)7	(4)7
1030917	1	1030887	TCTGGAACCCTTTCGGGTCGCCGGTTTT	*(serT)hyaA*	(274)0	(154)0
*1049918*	2	*1049983*	TAAGGAAATTGTTACGAAAGCTATTAAT	*insB-4/cspH* converg.	-	-
1129384	48	1129409	TAAAGATTACCCGTCCCTTGCCGATAAA	*flgMN*	451	318
1137565	47	1137556	TCAAGTCCGGCGGGTCGCTGCCGATAAT	*flgKL*	318	138
1241331	2	1241357	TAAGTAAAACGCTGTCTCTGCCGCTAAT	*cvrA*	43	28
1243779	67	1243800	TTAAGTTTTGTTAACTGTGACCGATAAA	*ycgR*	104	0*
1490461	23	1490443	TAAGTAATTACCGTCAAGTGCCGATGAC	*trg*	108	4*
1512636	3	1512671	GCTGGGAATAAACCATATTGCCGATAAA	*(ydcU)*	7	10
1644411	16	1644394	TAAAGATTTTTTTGTGCATGCCGATAGT	*flxA*	280	2*
1676085	1	1676104	TAATATTTTGCGAGTTCACGCCGAAATA	*pntA*	140	169
1823008	44	1823029	AACGTAAATCACCCGAGTTGCCGATAAC	*ves*	18	0
1840188	1	1840186	TAACGTTATTGTCTCTGCTACTGATAAC	*ynjH*	29	2*
1970737	57	1970761	TAAAGTTTCCCCCCTCCTTGCCGATAAC	*tar-tap-cheRBYZ*	251	0*
1975314	101	1975347	TAAACTTTCCCAGAATCCTGCCGATATT	*(flhC)motAB-cheAW*	(320)302	(107*)1*
1979379	2	1979356	TGAAATTGCACCAGATCGAGCCGATAAT	*(otsA)*	26	27
1999845	91	1999853	TGCAGAAACGGATAATCATGCCGATAAC	*fliAYZ*	1257	15*
2001692	110	2001721	TAAAGGTTGTTTTACGACAGACGATAAC	*fliC*	2319	11*
2001856	43	2001841	TAAACTTTGCGCAATTCAGACCGATAAC	*fliDST*	633	356
2017629	18	2017601	TCAAGACGCAGGATAATTAGCCGATAAG	*fliLMNOPQR*	989	980
2232326	3	2232358	TAACAAAACGCTGTAAGCGGCCGATATC	*(preT)*	49	47
2484776	2	2484747	TCAACTTCAACCACAATGGGTCGATATC	*(evgS)*	23	33
2683494	2	2683512	AAAGCGTGAAATGAACATTGCCGATTAT	*(glyA)*	375	415
2850743	51	2850785	TAAAATTATAGGCGTCGGTGCCGATAAC	*(hypD)*	6	7
2860228	2	2860201	TAAGGATCTTGGTCTGGTTGCCGATACA	*(ygbJ)ygbK*	(12)13	(10)10
3082647	11	3082628	TAAAGATGCCGGAAGAGTAGCCGATATG	*(speA)*	133	193
3101896	3	3101871	GGCGCAACGGCAGATTGCTGCCGATAAC	*(mutY)yggX*	(128)369	(104)370
3217148	21	3217162	TAAAGATAACCGCAGCGGGGCCGACATA	*aer*	225	12*
3246148	2	3246175	AAAGCGACCAATTAACAGCGCCGATAAA	*(yqjA)*	140	130
3339939	2	3339902	TAAACTTCTGCTGCGCGTAAACGATATT	*(kdsD)kdsC*	(158)228	(155)241
3524439	9	3524407	CAAGTTAAACTCCACGCTTGCCGATAGC	*yrfF*	62	53
3677244	67	3677273	TAAAGTTCTGCCCTTACGCGCCGATAAT	*yhjH*	511	2*
*3706702*	1	*3706643*	TCCTCTATCACCGACCAAATTCGAAAAG	*(proK)*	62	44
3844022	11	3844039	TAAAAAAGCGATTGGCGCTGCCGATGGT	*(uhpT)*	25	1
3846459	1	3846440	AAAACAGGGTCGCTAACAGGCCGATATC	*(uhpC)*	9	8
4016533	1	4016575	GAGAGTTTTTTCATTGCCTGCCGATAAT	*(rmuC)*	60	78
4119023	1	4118999	TACAGATTTTGTCGATTTCGTCGATAAA	*(hslU)*	1085	1337
4131349	3	4131304	TAAACAGGCGAAGAAATTTGCCGATATG	*(metF)*	6	3
4162805	3	4162795	TGAAGGCGCAGCACGCAGTGACGATAAC	*(btuB)*	72	92
4228823	6	4228793	GAAAGAGTATCTGGTGACGGTCGATAAA	*(rluF)*	73	73
4327161	19	4327146	TAAAGTTCTGGCAGAGCAGGTCGATGAA	*(yjdA)yjcZ*	(111)52	(11*)1*
4564123	1	4564096	GAATAAACTGCAGATCTTTGCCGATATT	*(yjiN)*	17	17
4589660	94	4589638	TAAAGTTTTTCCTTTCCAGGCCGAAAAT	*tsr*	630	9*
4591362	3	4591380	AAAGATTAATCTCCTTATGCCCGATAAC	*tsr/yjiZ* convergent	-	-
4621181	1	4621145	TACAGCCCCCGCCATCCATGCCGATAAC	*(lplA)*	30	31

1 Normalized gene expression values generated by Rockhopper. Expression values are for first gene in operon. Values in parentheses correspond with gene name in parentheses.

2 Peak centers and motif centers refer to genome coordinates relative to NC_000913.2.

3 Peak centers and motif centers in italics are likely false positives, based on the location of the motif relative to the peak center.

4 Fold Above Threshold (FAT).

5 Gene(s) adjacent to binding site. Parentheses indicate an intragenic binding site, while those that are underlined are in the antisense orientation and those not underlined are in the sense orientation.

6 Asterisks indicate significant differential expression (as defined in Methods) between motile MG1655 and Δ*fliA*.
