# Correction: Comprehensive Mapping of the *Escherichia coli* Flagellar Regulatory Network

**DOI:** 10.1371/journal.pgen.1005456

**Published:** 2015-09-17

**Authors:** Devon M. Fitzgerald, Richard P. Bonocora, Joseph T. Wade

The email address for the corresponding author is incorrect. The correct email address is: joseph.wade@health.ny.gov.

The section entitled “ChIP-seq data analysis” in the Materials and Methods section mentions reference [54]. This is incorrect. Instead reference [63] should be cited. The correct text is included here:

Once T1 and T2 were chosen, peak calling was performed as previously described (Supplementary Material of [63]). Briefly, a region was identified as a peak if both replicates showed enrichment above the corresponding thresholds for each strand. For a peak to be called there must be a peak on the plus strand within a threshold distance of a peak on the minus strand, as previously described (Supplementary Material of [63]).
