# Supplementary material for: Comprehensive Mapping of the Escherichia coli Flagellar Regulatory Network
Source: PLoS Genet. 2014 Oct 2;10(10):e1004649. doi: 10.1371/journal.pgen.1004649 (PMC4183435; doi:10.1371/journal.pgen.1004649)
Supplement: Figure S1 — Strain construction and validation. (A) Tag locations for internal 3×FLAG-tagging of FlhD and FlhC. Black lines represent unstructured regions, red cylinders represent α-helices, and blue boxes represent β-sheets. Gold stars represent Zn-binding cysteine residues. Insets show amino acid sequence surrounding tag insertion sites. (B) Location, identity, and direction of IS element insertions present in each motility-selected strain. The boxes represent regions that are duplicated during insertion. (C) Soft agar motility of motile MG1655 and strains in which a thyA cassette has been inserted in each of the IS element insertion locations described above. (D) Soft agar motility of motile MG1655 and epitope-tagged strains. (E) Soft agar motility of motile MG1655, isogenic deletions, and complemented strains. Note that ΔflhC+pflhC is non-motile due to disruption of the promoter of the motAB-cheAW operon. (PDF) [file pgen.1004649.s001.pdf]

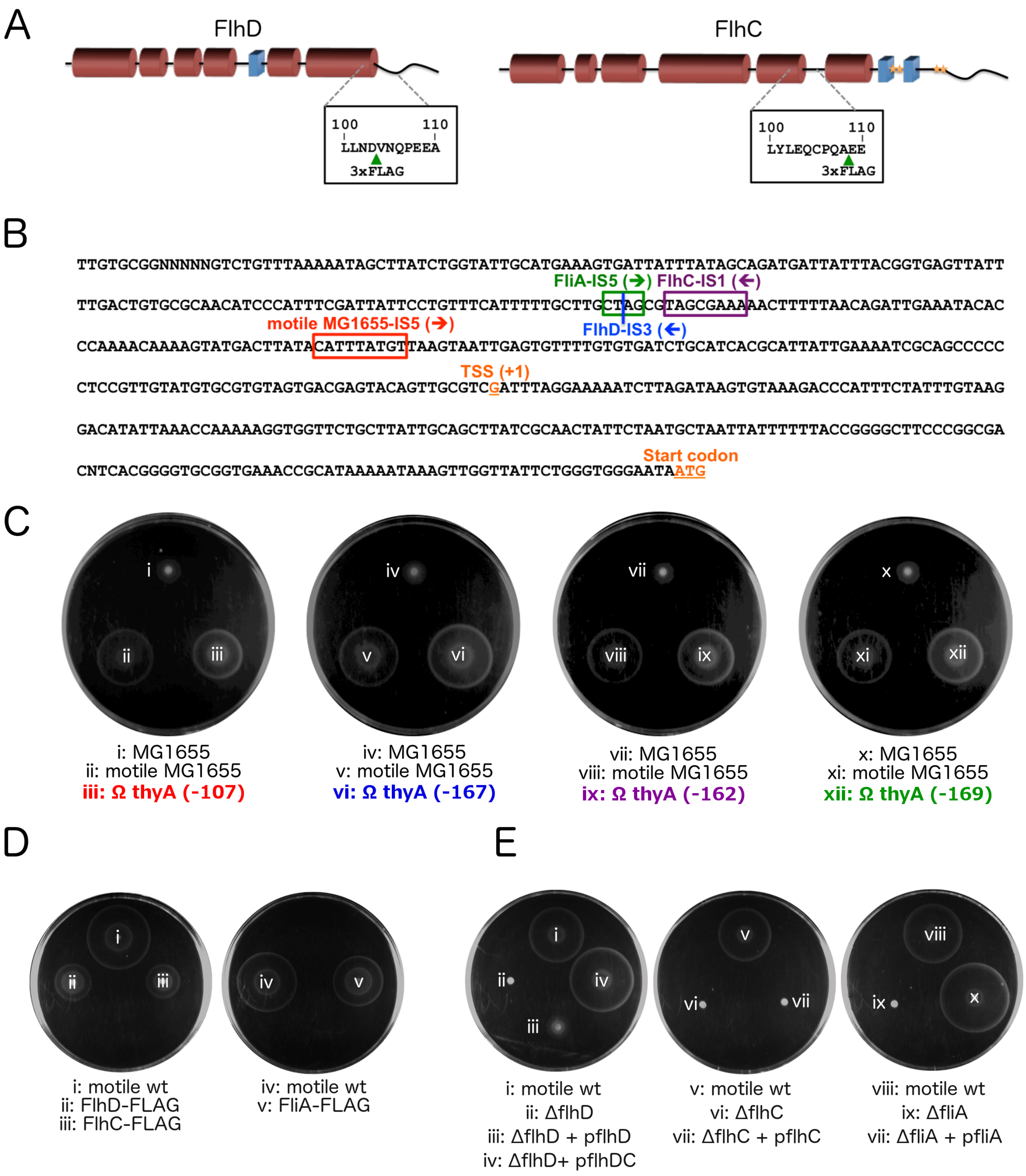

A
FlhD
100
110
LLNDVNQPEEA
3xFLAG
FlhC
100
110
LYLEQCPQAEE
3xFLAG
B
TTGTGCGGNNNNNGTCTGTTTAAAAATAGCTTATCTGGTATTGCATGAAAGTGATTATTTATAGCAGATGATTATTTACGGTGAGTTATT
FliA-IS5 (→) FlhC-IS1 (←)
TTGACTGTGCGCAACATCCCATTTCGATTATTCCTGTTTCATTTTTGCTTGCTAGCGTAGCGAAAAACTTTTTAACAGATTGAAATACAC
motile MG1655-IS5 (→)
FlhD-IS3 (←)
CCAAAACAAAAGTATGACTTATACATTTATGTTAAGTAATTGAGTGTTTTGTGTGATCTGCATCACGCATTATTGAAAATCGCAGCCCCC
TSS (+1)
CTCCGTTGTATGTGCGTGTAGTGACGAGTACAGTTGCGTCGATTTAGGAAAAATCTTAGATAAGTGTAAAGACCCATTTCTATTTGTAAG
GACATATTAAACCAAAAAGGTGGTTCTGCTTATTGCAGCTTATCGCAACTATTCTAATGCTAATTATTTTTTACCGGGCTTCCCGGCGA
Start codon
CNTCACGGGGTGCGGTGAAACCGCATAAAAATAAAGTTGGTTATTCTGGGTGGGAATAATG
C
i
ii
iii
iv
v
vi
vii
viii
ix
x
xi
xii
i: MG1655
ii: motile MG1655
iii: Ω thyA (-107)
iv: MG1655
v: motile MG1655
vi: Ω thyA (-167)
vii: MG1655
viii: motile MG1655
ix: Ω thyA (-162)
x: MG1655
xi: motile MG1655
xii: Ω thyA (-169)
D
i
ii
iii
iv
v
i: motile wt
ii: FlhD-FLAG
iii: FlhC-FLAG
iv: motile wt
v: FliA-FLAG
E
i
ii
iii
iv
v
vi
vii
viii
ix
x
i: motile wt
ii: ΔflhD
iii: ΔflhD + pflhD
iv: ΔflhD+ pflhDC
v: motile wt
vi: ΔflhC
vii: ΔflhC + pflhC
viii: motile wt
ix: ΔfliA
vii: ΔfliA + pfliA
