# Supplementary figures and images for: Comprehensive Mapping of the Escherichia coli Flagellar Regulatory Network
Source: PLoS Genet. 2014 Oct 2;10(10):e1004649. doi: 10.1371/journal.pgen.1004649 (PMC4183435; doi:10.1371/journal.pgen.1004649)

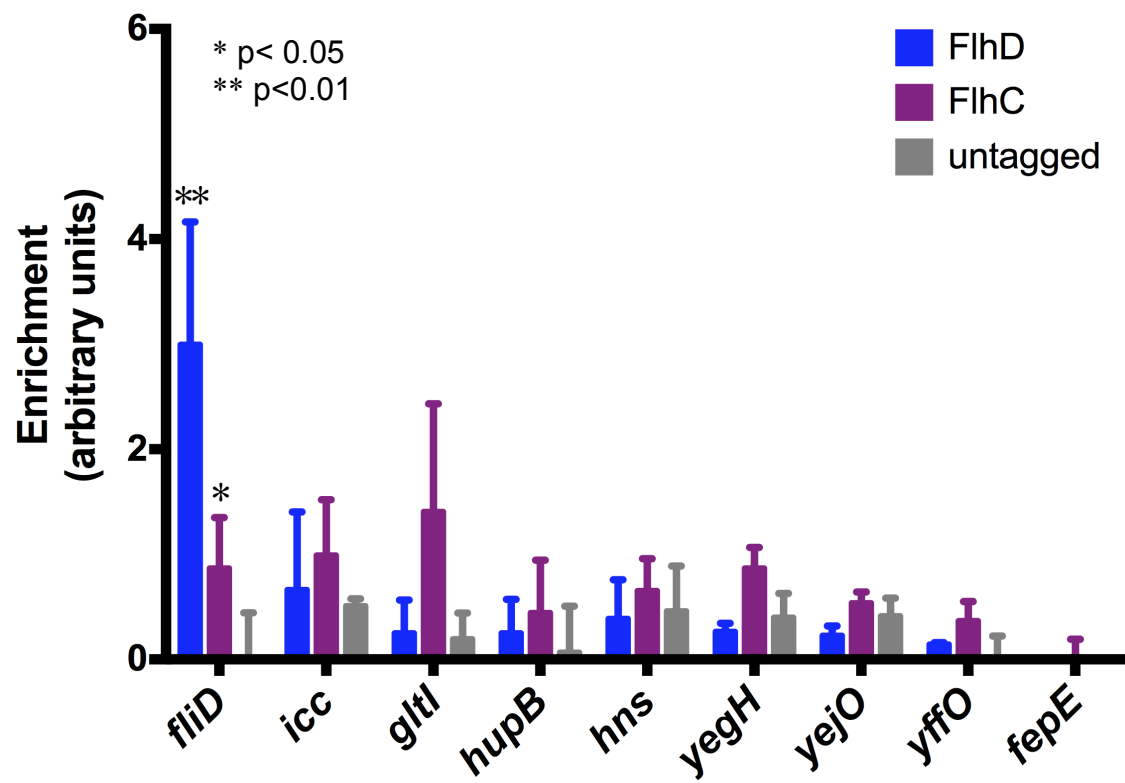

Supplement: Figure S2 — Presence of FlhDC motif and in vitro binding is not predictive of in vivo binding. Stafford et al. [25] predicted FlhDC binding sites based on the consensus motif of characterized binding sites. The sites shown in this figure had good matches to the consensus and demonstrated weak in vitro binding. With the exception of fliD and yecR (not shown), none of the predicted sites showed in vivo FlhDC binding in targeted ChIP-qPCR assays (n = 4, * p<0.05, ** p<0.01). (PDF) [file pgen.1004649.s002.pdf]

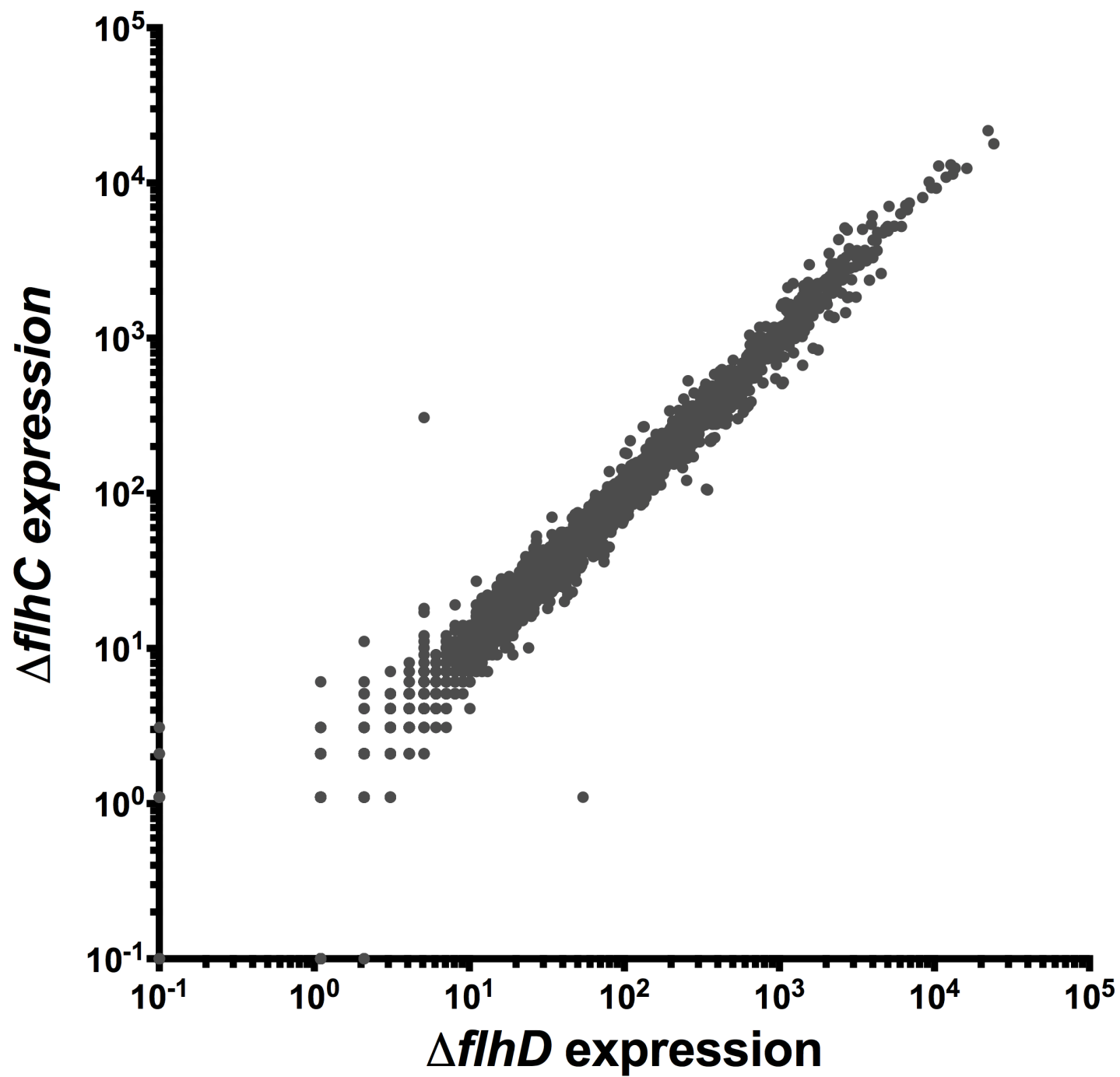

Supplement: Figure S3 — Genome-wide expression in Δ flhD and Δ flhC . Expression of all genes in ΔflhD versus ΔflhC. Gene expression values represent normalized expression values calculated by Rockhopper. (PDF) [file pgen.1004649.s003.pdf]

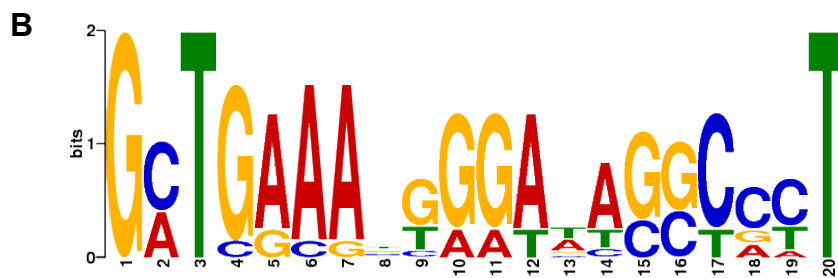

Supplement: Figure S4 — Comparison of FliA motifs from this study and Cho et al. [55] . Motifs were generated from sequence surrounding binding sites identified only in our study (n = 27) or only in Cho et al. (n = 29). (A) FliA binding sites unique to our study yielded a highly significant motif (27/30 sites, E-value = 1.5e-27) similar to that described for FliA. (B) The best-scoring motif for FliA binding regions unique to Cho et al is not significantly enriched, and shows no similarity to described FliA motifs (best-scoring motif: 10/29, E-value = 3.5). It should also be noted that Cho et al failed to detect some well-characterized FliA promoters such as those upstream of fliAZY and fliC. (PDF) [file pgen.1004649.s004.pdf]

A

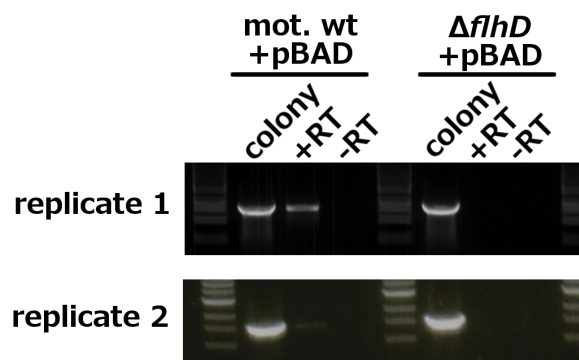

B

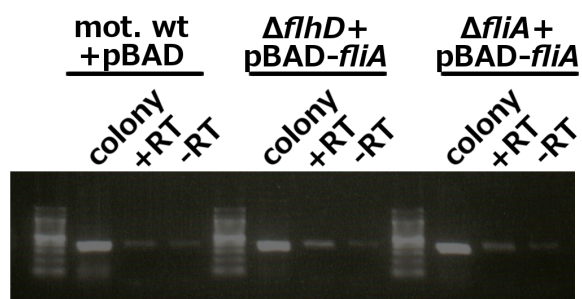

Supplement: Figure S5 — flgJK is dual regulated but flgBAE is not. (A) RT-PCR using an upstream primer within flgJ and a downstream primer within flgK yielded a band of the expected size in motile MG1655, but not in ΔflhD. This confirms that flgKL can be transcribed as part of the upstream FlhDC-dependent operon. Lanes labeled “colony” are a colony (genomic DNA) PCR control, “+RT” are RT-PCR, and “−RT” are controls in which no reverse transcriptase was added during cDNA synthesis. (B) RT-PCR using an upstream primer within cheZ and a downstream primer within flhBAE yielded very little product. Product slightly increased, relative to the −RT control, when fliA was overexpressed. This small amount of read-through at very high FliA levels is unlikely to be physiologically relevant. (PDF) [file pgen.1004649.s005.pdf]

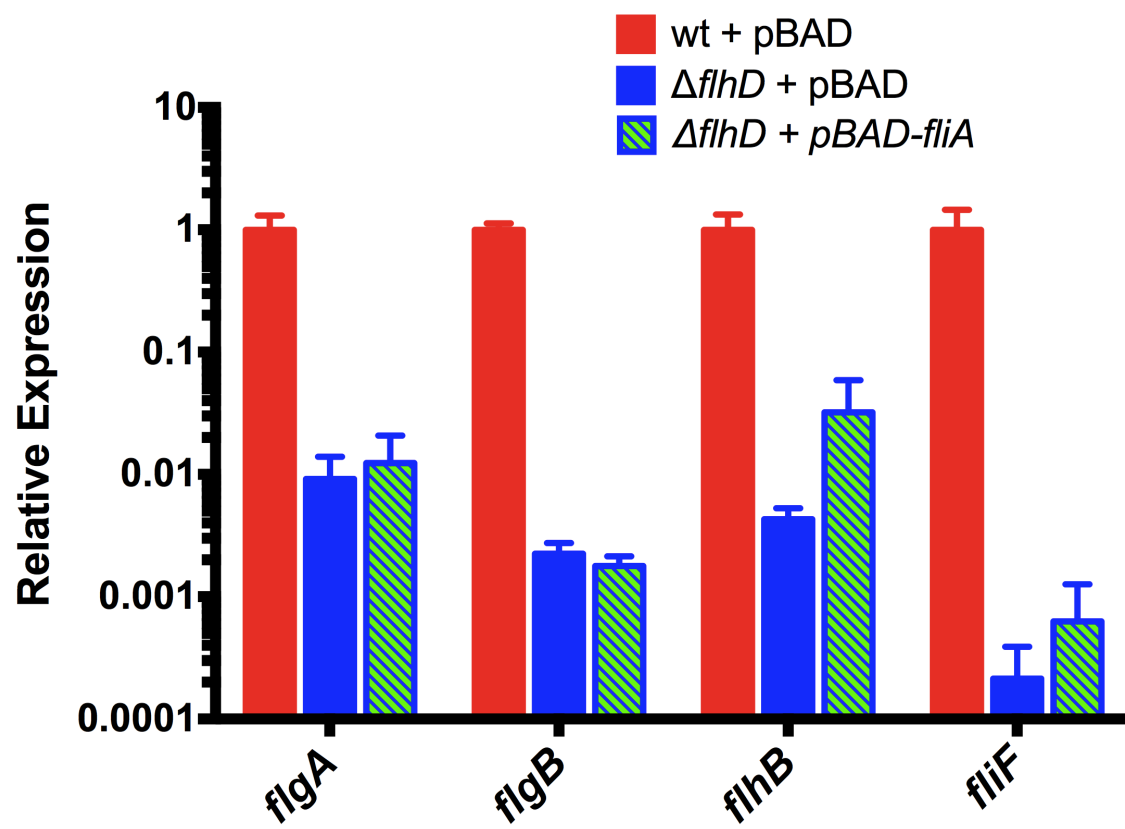

Supplement: Figure S6 — All class 2 genes are not transcribed by FliA. Expression of flgA, flgB, and fliF cannot be rescued by overexpression FliA in ΔflhD. Expression of flhB is moderately increased in the ΔflhD+pBAD-fliA strain, potentially due to read-through from the upstream FliA-dependent tar-tap-cheRBYZ (see Figure S4). (PDF) [file pgen.1004649.s006.pdf]

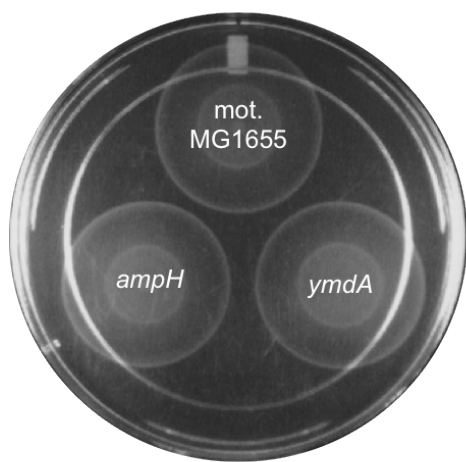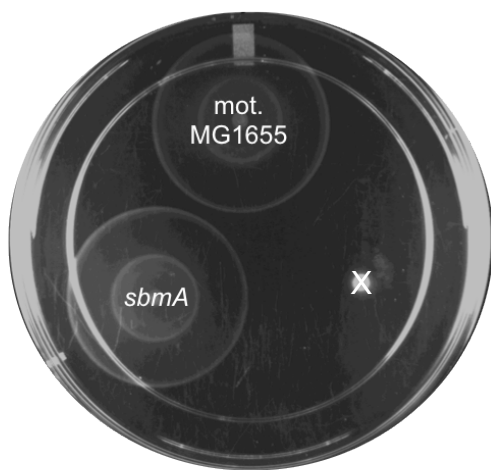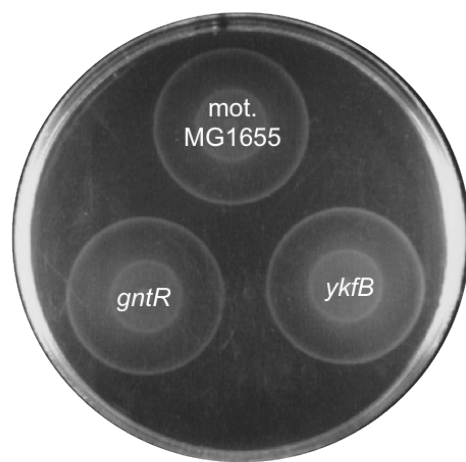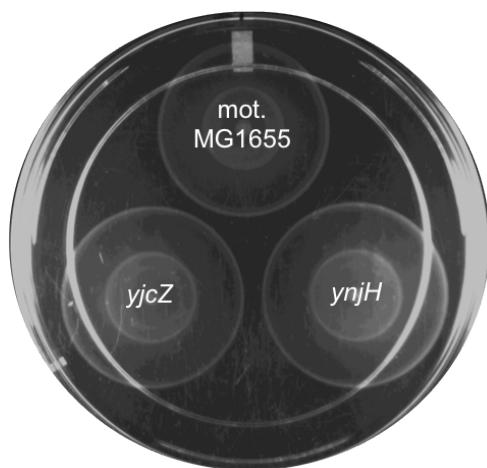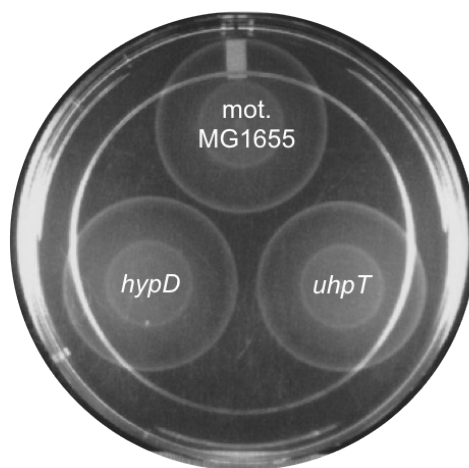

Supplement: Figure S7 — Novel FlhDC and FliA targets are not required for motility. Soft agar motility of motile MG1655 and single gene deletions of FlhDC and FliA target genes. Images are representative of 5 biological replicates per strain. (PDF) [file pgen.1004649.s007.pdf]
