# Supplementary material for: Comprehensive Mapping of the Escherichia coli Flagellar Regulatory Network
Source: PLoS Genet. 2014 Oct 2;10(10):e1004649. doi: 10.1371/journal.pgen.1004649 (PMC4183435; doi:10.1371/journal.pgen.1004649)
Supplement: File S1 — Sequence of flhDC locus in motile MG1655 and epitope-tagged strains. (DOCX) [file pgen.1004649.s015.docx]

**Supplemental File S1**: **Sequence of the *flhDC* locus in motile MG1655 and epitope-tagged strains.** Each sequence is labeled with the strain name and description. IS elements are enclosed in square brackets, bolded, and highlighted in grey. Duplicated chromosomal sequence (if present) is underlined and highlighted in yellow. The primary TSS (genome position 1976419 in NC_000913.2) and start codons are bolded, underlined, and highlighted in green. Stop codons are bolded, underlined, and highlighted in red. The *flhD* open reading frame (ORF) is denoted by bolded, blue text. The *flhC* ORF is denoted by bolded, purple text. Epitope tags are highlighted in the color of the ORF in which they reside (blue for *flhD*, purple for *flhC*).

**DMF36 (motile MG1655 with IS1 upstream of -107)**

TATAGCAGATGATTATTTACGGTGAGTTATTTTGACTGTGCGCAACATCCCATTTCGATTATTCCTGTTTCATTTTTGCTTGCTAGCGTAGCGAAAAACTTTTTAACAGATTGAAATACACCCAAAACAAAAGTATGACTTATACATTTATGT**[GGTGATGCTGCCAACTTACTGATTTAGTGTATGATGGTGTTTTTGAGGTGCTCCAGTGGCTTCTGTTTCTATCAGCTGTCCCTCCTGTTCAGCTACTGACGGGGTGGTGCGTAACGGCAAAAGCACCGCCGGACATCAGCGCTATCTCTGCTCTCACTGCCGTAAAACATGGCAACTGCAGTTCACTTACACCGCTTCTCAACCCGGTACGCACCAGAAAATCATTGATATGGCCATGAATGGCGTTGGATGCCGGGCAACAGCCCGCATTATGGGCGTTGGCCTCAACACGATTTTACGTCACTTAAAAAACTCAGGCCGCAGTCGGTAACCTCGCGCATACAGCCGGGCAGTGACGTCATCGTCTGCGCGGAAATGGACGAACAGTGGGGCTATGTCGGGGCTAAATCGCGCCAGCGCTGGCTGTTTTACGCGTATGACAGTCTCCGGAAGACGGTTGTTGCGCACGTATTCGGTGAACGCACTATGGCGACGCTGGGGCGTCTTATGAGCCTGCTGTCACCCTTTGACGTGGTGATATGGATGACGGATGGCTGGCCGCTGTATGAATCCCGCCTGAAGGGAAAGCTGCACGTAATCAGCAAGCGATATACGCAGCGAATTGAGCGGCATAACCTGAATCTGAGGCAGCACCTGGCACGGCTGGGACGGAAGTCGCTGTCGTTCTCAAAATCGGTGGAGCTGCATGACAAAGTCATCGGGCATTATCTGAACATAAAACACTATCAATAAGTTGGAGTCATTACC]**CATTTATGTTAAGTAATTGAGTGTTTTGTGTGATCTGCATCACGCATTATTGAAAATCGCAGCCCCCCTCCGTTGTATGTGCGTGTAGTGACGAGTACAGTTGCGTC**G**ATTTAGGAAAAATCTTAGATAAGTGTAAAGACCCATTTCTATTTGTAAGGACATATTAAACCAAAAAGGTGGTTCTGCTTATTGCAGCTTATCGCAACTATTCTAATGCTAATTATTTTTTACCGGGGCTTCCCGGCGACATCACGGGGTGCGGTGAAACCGCATAAAAATAAAGTTGGTTATTCTGGGTGGGAATA**ATGCATACCTCCGAGTTGCTGAAACACATTTATGACATCAACTTGTCATATTTACTACTTGCACAGCGTTTGATTGTTCAGGACAAAGCGTCCGCTATGTTTCGTCTCGGCATAAATGAAGAAATGGCGACAACGTTAGCGGCACTGACTCTTCCGCAAATGGTTAAGCTGGCAGAAACCAATCAACTGGTTTGTCACTTCCGTTTTGACAGCCACCAGACGATTACTCAGTTGACGCAAGATTCCCGCGTTGACGATCTCCAGCAAATTCATACCGGCATCATGCTCTCAACACGCTTGCTGAATGATGTTAATCAGCCTGAAGAAGCGCTGCGCAAGAAAAGGGCCTGA**TC**ATGAGTGAAAAAAGCATTGTTCAGGAAGCGCGGGATATTCAGCTGGCAATGGAATTGATCACCCTGGGCGCTCGTTTGCAGATGCTGGAAAGCGAAACACAGTTAAGTCGCGGACGCCTGATAAAACTTTATAAAGAACTGCGCGGAAGCCCACCGCCGAAAGGCATGCTGCCATTCTCAACCGACTGGTTTATGACCTGGGAACAAAACGTTCATGCTTCGATGTTCTGTAATGCATGGCAGTTTTTACTGAAAACCGGTTTGTGTAATGGCGTCGATGCGGTGATCAAAGCCTACCGTTTATACCTTGAACAGTGCCCACAAGCAGAAGAAGGACCACTGCTGGCATTAACCCGTGCCTGGACATTGGTGCGGTTTGTTGAAAGTGGATTACTGCAACTTTCCAGCTGCAACTGCTGCGGCGGCAATTTTATTACCCACGCTCACCAGCCTGTTGGCAGCTTTGCCTGCAGCTTATGTCAACCGCCATCCCGGGCAGTAAAAAGACGTAAACTTTCCCAGAATCCTGCCGATATTATCCCACAACTGCTGGATGAACAGAGAGTACAGGCTGTTTAA**

**DMF14 (FlhD-FLAG with IS3 upstream of -167)**

TATAGCAGATGATTATTTACGGTGAGTTATTTTGACTGTGCGCAACATCCCATTTCGATTATTCCTGTTTCATTTTTGCTTGCT**[TGATCCTACCCACGTAATATGGACACAGGCCTAAGCGAGGTTCTTGTTTTCAAATTGTTCCGGACTGAGGCCGCCACACCAACTGTGCCGCCGCCACCGATTGTAATCACATTCGATATAATTAAACACCGTTGCCCGCATTATTTCCCGGCTGATAAAGTGTTCTCCATGGATACATTCCACTTTCAGCGAATGAAAGAAGCTTTCCACGCAGGCATTATCGTAGCAGCAACCTTTTGCGCTCATACTTCCACGCAGATTATGCCGCTTCAGTTGCGCCTGATAATCTGCTGAACAGTACTGGCCTCCACGGTCCGTGTGAACGATAACGTTCCGGGGCCTCTTACGCCGCCACAGCGCCATCTGCAGGGCATCGCAGGCCAGTTGCGCCGTCATGCGTGGCGACATTGACCAGCCAATAACGGCACGTGACCACAGGTCAATGACCACTGCCAGATACAGCCAGCCTTCATCTGTACGTAAGTACGTGATGTCTCCTGCCCACTTCTGGTTCGGGCCACTGGCGTAAAAATCCTGCTCCAACAGATTTTCTGACACAGGCAGGCCGTGTGCGCGGTAGCTGACCGGGCTGAACTTCCGGGAGGCCTTTGCCCTCAGTCCCTGACGGCGCAGGCTTGCCGCCACGGTTTTTACGTTAAAGGGGTAACCCTGAGCACGCAGTTCATCCGTCAGGCGTGGGGCACCGTAACGCTGTTTTGACCGGGTAAAAGCCGCGAGGACAACGCTGTCGCAGTGTTGGCGGAACTGCTGACGCGTGCTTATCCTTGTCCGCCGCTGACACCACGTATACCAGCCGCTGCGGGCCACCCGGAGCACGCGGCACATTGCTTTGATGCTGAACTCAGCCTGATGTTTTTCAATAAAGACATACTTCATTTCAGGCGCTTCGCGAAGTATGTCGCGGCCTTTTGGAGGATAGCCAGCTCTTCATCCCGTTCTGCCAGCTGGCGTTTGAGACGTGCAATCTCGGTAGACATCTCCAGTTCACGTTCAGAAGACGTCTGCTGATTTTGCTGTTTACTGCGCCAGTTGTAGAGTTGTGATTCATACAGGCTGAGTTCACGGGCTGCGGCAGTAACACCGATGCGTTCAGCAAGCTTCAGGGCTTCACTGCGAAATTCAGGCGAATGCTGTTTACGGGGTTTTTTACTGGTTGATACTGTTTTTGTCATGTGAGTCACCTCTGACTGAGAGTTTACTCACTTAGCCGCGTGTCCACTATTGCTGGGTAAGATCAGAT]**AGCGTAGCGAAAAACTTTTTAACAGATTGAAATACACCCAAAACAAAAGTATGACTTATACATTTATGTTAAGTAATTGAGTGTTTTGTGTGATCTGCATCACGCATTATTGAAAATCGCAGCCCCCCTCCGTTGTATGTGCGTGTAGTGACGAGTACAGTTGCGTC**G**ATTTAGGAAAAATCTTAGATAAGTGTAAAGACCCATTTCTATTTGTAAGGACATATTAAACCAAAAAGGTGGTTCTGCTTATTGCAGCTTATCGCAACTATTCTAATGCTAATTATTTTTTACCGGGGCTTCCCGGCGACATCACGGGGTGCGGTGAAACCGCATAAAAATAAAGTTGGTTATTCTGGGTGGGAATA**ATGCATACCTCCGAGTTGCTGAAACACATTTATGACATCAACTTGTCATATTTACTACTTGCACAGCGTTTGATTGTTCAGGACAAAGCGTCCGCTATGTTTCGTCTCGGCATAAATGAAGAAATGGCGACAACGTTAGCGGCACTGACTCTTCCGCAAATGGTTAAGCTGGCAGAAACCAATCAACTGGTTTGTCACTTCCGTTTTGACAGCCACCAGACGATTACTCAGTTGACGCAAGATTCCCGCGTTGACGATCTCCAGCAAATTCATACCGGCATCATGCTCTCAACACGCTTGCTGAATGATGGCGGTGGCGACTACAAAGACCATGACGGTGATTATAAAGATCATGACATCGACTACAAGGATGACGATGACAAGGTTAATCAGCCTGAAGAAGCGCTGCGCAAGAAAAGGGCCTGA**TC**ATGAGTGAAAAAAGCATTGTTCAGGAAGCGCGGGATATTCAGCTGGCAATGGAATTGATCACCCTGGGCGCTCGTTTGCAGATGCTGGAAAGCGAAACACAGTTAAGTCGCGGACGCCTGATAAAACTTTATAAAGAACTGCGCGGAAGCCCACCGCCGAAAGGCATGCTGCCATTCTCAACCGACTGGTTTATGACCTGGGAACAAAACGTTCATGCTTCGATGTTCTGTAATGCATGGCAGTTTTTACTGAAAACCGGTTTGTGTAATGGCGTCGATGCGGTGATCAAAGCCTACCGTTTATACCTTGAACAGTGCCCACAAGCAGAAGAAGGACCACTGCTGGCATTAACCCGTGCCTGGACATTGGTGCGGTTTGTTGAAAGTGGATTACTGCAACTTTCCAGCTGCAACTGCTGCGGCGGCAATTTTATTACCCACGCTCACCAGCCTGTTGGCAGCTTTGCCTGCAGCTTATGTCAACCGCCATCCCGGGCAGTAAAAAGACGTAAACTTTCCCAGAATCCTGCCGATATTATCCCACAACTGCTGGATGAACAGAGAGTACAGGCTGTTTAA**

**DMF11 (FlhC-FLAG with IS1 upstream of -162)**

TATAGCAGATGATTATTTACGGTGAGTTATTTTGACTGTGCGCAACATCCCATTTCGATTATTCCTGTTTCATTTTTGCTTGCTAGCGTAGCGAAA**[GGTAATGACTCCAACTTATTGATAGTGTTTTATGTTCAGATAATGCCCGATGACTTTGTCATGCAGCTCCACCGATTTTGAGAACGACAGCGACTTCCGTCCCAGCCGTGCCAGGTGCTGCCTCAGATTCAGGTTATGCCGCTCAATTCGCTGCGTATATCGCTTGCTGATTACGTGCAGCTTTCCCTTCAGGCGGGATTCATACAGCGGCCAGCCATCCGTCATCCATATCACCACGTCAAAGGGTGACAGCAGGCTCATAAGACGCCCCAGCGTCGCCATAGTGCGTTCACCGAATACGTGCGCAACAACCGTCTTCCGGAGACTGTCATACGCGTAAAACAGCCAGCGCTGGCGCGATTTAGCCCCGACATAGCCCCACTGTTCGTCCATTTCCGCGCAGACGATGACGTCACTGCCCGGCTGTATGCGCGAGGTTACCGACTGCGGCCTGAGTTTTTTAAGTGACGTAAAATCGTGTTGAGGCCAACGCCCATAATGCGGGCTGTTGCCCGGCATCCAACGCCATTCATGGCCATATCAATGATTTTCTGGTGCGTACCGGGTTGAGAAGCGGTGTAAGTGAACTGCAGTTGCCATGTTTTACGGCAGTGAGAGCAGAGATAGCGCTGATGTCCGGCGGTGCTTTTGCCGTTACGCACCACCCCGTCAGTAGCTGAACAGGAGGGACAGCTGATAGAAACAGAAGCCACTGGAGCNNCTCAAAAACACCATCATACACTAAATCAGTAAGTTGGCAGCATCACC]**TAGCGAAAAACTTTTTAACAGATTGAAATACACCCAAAACAAAAGTATGACTTATACATTTATGTTNAGTAATTGAGTGTTTTGTGTGATCTGCATCACGCATTATTGAAAATCGCAGCCCCCCTCCGTTGTATGTGCGTGTAGTGACGAGTACAGTTGCGTC**G**ATTTAGGAAAAATCTTAGATAAGTGTAAAGACCCATTTCTATTTGTAAGGACATATTAAACCAAAAAGGTGGTTCTGCTTATTGCAGCTTATCGCAACTATTCTAATGCTAATTATTTTTTACCGGGGCTTCCCGGCGACATCACGGGGTGCGGTGAAACCGCATAAAAATAAAGTTGGTTATTCTGGGTGGGAATA**ATGCATACCTCCGAGTTGCTGAAACACATTTATGACATCAACTTGTCATATTTACTACTTGCACAGCGTTTGATTGTTCAGGACAAAGCGTCCGCTATGTTTCGTCTCGGCATAAATGAAGAAATGGCGACAACGTTAGCGGCACTGACTCTTCCGCAAATGGTTAAGCTGGCAGAAACCAATCAACTGGTTTGTCACTTCCGTTTTGACAGCCACCAGACGATTACTCAGTTGACGCAAGATTCCCGCGTTGACGATCTCCAGCAAATTCATACCGGCATCATGCTCTCAACACGCTTGCTGAATGATGTTAATCAGCCTGAAGAAGCGCTGCGCAAGAAAAGGGCCTGA**TC**ATGAGTGAAAAAAGCATTGTTCAGGAAGCGCGGGATATTCAGCTGGCAATGGAATTGATCACCCTGGGCGCTCGTTTGCAGATGCTGGAAAGCGAAACACAGTTAAGTCGCGGACGCCTGATAAAACTTTATAAAGAACTGCGCGGAAGCCCACCGCCGAAAGGCATGCTGCCATTCTCAACCGACTGGTTTATGACCTGGGAACAAAACGTTCATGCTTCGATGTTCTGTAATGCATGGCAGTTTTTACTGAAAACCGGTTTGTGTAATGGCGTCGATGCGGTGATCAAAGCCTACCGTTTATACCTTGAACAGTGCCCACAAGCAGAAGAAGGCGGTGGCGACTACAAAGACCATGACGGTGATTATAAAGATCATGACATCGACTACAAGGATGACGATGACAAGGGACCACTGCTGGCATTAACCCGTGCCTGGACATTGGTGCGGTTTGTTGAAAGTGGATTACTGCAACTTTCCAGCTGCAACTGCTGCGGCGGCAATTTTATTACCCACGCTCACCAGCCTGTTGGCAGCTTTGCCTGCAGCTTATGTCAACCGCCATCCCGGGCAGTAAAAAGACGTAAACTTTCCCAGAATCCTGCCGATATTATCCCACAACTGCTGGATGAACAGAGAGTACAGGCTGTTTAA**CAACAGNGGAAGGANGATGTCGTGC

**RPB081 (FliA-FLAG with IS5 upstream of -169)**

TATAGCAGATGATTATTTACGGTGAGTTATTTTGACTGTGCGCAACATCCCATTTCGATTATTCCTGTTTCATTTTTGCTTGCTAG**[GGAAGGTGCGAATAAGCGGGGAAATTCTTCTCGGCTGACTCAGTCATTTCATTTCTTCATGTTTGAGCCGATTTTTTCTCCCGTAAATGCCTTGAATCAGCCTATTTAGACCGTTTCTTCGCCATTTAAGGCGTTATCCCCAGTTTTTAGTGAGATCTCTCCCACTGACGTATCATTTGGTCCGCCCGAAACAGGTTGGCCAGCGTGAATAACATCGCCAGTTGGTTATCGTTTTTCAGCAACCCCTTGTATCTGGCTTTCACGAAGCCGAACTGTCGCTTGATGATGCGAAATGGGTGCTCCACCCTGGCCCGGATGCTGGCTTTCATGTATTCGATGTTGATGGCCGTTTTGTTCTTGCGTGGATGCTGTTTCAAGGTTCTTACCTTGCCGGGGCGCTCGGCGATCAGCCAGTCCACATCCACCTCGGCCAGCTCCTCGCGCTGTGGCGCCCCTTGGTAGCCGGCATCGGCTGAGACAAATTGCTCCTCTCCATGCAGCAGATTACCCAGCTGATTGAGGTCATGCTCGTTGGCCGCGGTGGTGACCAGGCTGTGGGTCAGGCCACTCTTGGCATCGACACCAATGTGGGCCTTCATGCCAAAGTGCCACTGATTGCCTTTCTTGGTCTGATGCATCTCCGGATCGCGTTGCTGCTCTTTGTTCTTGGTCGAGCTGGGTGCCTCAATGATGGTGGCATCGACCAAGGTGCCTTGAGTCATCATGACGCCTGCTTCGGCCAGCCAGCGATTGATGGTCTTGAACAATTGGCGGGCCAGTTGATGCTGCTCCAGCAGGTGGCGGAAATTCATGATGGTGGTGCGGTCCGGCAAGGCGCTATCCAGGGATAACCGGGCAAACAGACGCATGGAGGCGATTTCGTACAGAGCATCTTCCATCGCGCCATCGCTCAGGTTGTACCAATGCTGCATGCAGTGAATGCGTAGCATGGTTTCCAGCGGATAAGGTCGCCGGCCATTACCAGCCTTGGGGTAAAACGGCTCGATGACTTCCACCATGTTTTGCCATGGCAGAATCTGCTCCATGCGGGACAAGAAAATCTCTTTTCTGGTCTGACGGCGCTTACTGCTGAATTCACTGTCGGCGAAGGTAAGTTGATGACTCATGATGAACCCTGTTCTATGGCTCCAGATGACAAACATGATCTCATATCAGGGACTTGTTCGCACCTTCC]**CTAGCGTAGCGAAAAACTTTTTAACAGATTGAAATACACCCAAAACAAAAGTATGACTTATACATTTATGTTAAGTAATTGAGTGTTTTGTGTGATCTGCATCACGCATTATTGAAAATCGCAGCCCCCCTCCGTTGTATGTGCGTGTAGTGACGAGTACAGTTGCGTC**G**ATTTAGGAAAAATCTTAGATAAGTGTAAAGACCCATTTCTATTTGTAAGGACATATTAAACCAAAAAGGTGGTTCTGCTTATTGCAGCTTATCGCAACTATTCTAATGCTAATTATTTTTTACCGGGGCTTCCCGGCGACATCACGGGGTGCGGTGAAACCGCATAAAAATAAAGTTGGTTATTCTGGGTGGGAATA**ATGCATACCTCCGAGTTGCTGAAACACATTTATGACATCAACTTGTCATATTTACTACTTGCACAGCGTTTGATTGTTCAGGACAAAGCGTCCGCTATGTTTCGTCTCGGCATAAATGAAGAAATGGCGACAACGTTAGCGGCACTGACTCTTCCGCAAATGGTTAAGCTGGCAGAAACCAATCAACTGGTTTGTCACTTCCGTTTTGACAGCCACCAGACGATTACTCAGTTGACGCAAGATTCCCGCGTTGACGATCTCCAGCAAATTCATACCGGCATCATGCTCTCAACACGCTTGCTGAATGATGTTAATCAGCCTGAAGAAGCGCTGCGCAAGAAAAGGGCCTGA**TC**ATGAGTGAAAAAAGCATTGTTCAGGAAGCGCGGGATATTCAGCTGGCAATGGAATTGATCACCCTGGGCGCTCGTTTGCAGATGCTGGAAAGCGAAACACAGTTAAGTCGCGGACGCCTGATAAAACTTTATAAAGAACTGCGCGGAAGCCCACCGCCGAAAGGCATGCTGCCATTCTCAACCGACTGGTTTATGACCTGGGAACAAAACGTTCATGCTTCGATGTTCTGTAATGCATGGCAGTTTTTACTGAAAACCGGTTTGTGTAATGGCGTCGATGCGGTGATCAAAGCCTACCGTTTATACCTTGAACAGTGCCCACAAGCAGAAGAAGGACCACTGCTGGCATTAACCCGTGCCTGGACATTGGTGCGGTTTGTTGAAAGTGGATTACTGCAACTTTCCAGCTGCAACTGCTGCGGCGGCAATTTTATTACCCACGCTCACCAGCCTGTTGGCAGCTTTGCCTGCAGCTTATGTCAACCGCCATCCCGGGCAGTAAAAAGACGTAAACTTTCCCAGAATCCTGCCGATATTATCCCACAACTGCTGGATGAACAGAGAGTACAGGCTGTTTAA**
